# Carbon Dots: Classification, Properties, Synthesis, Characterization, and Applications in Health Care—An Updated Review (2018–2021)

**DOI:** 10.3390/nano11102525

**Published:** 2021-09-27

**Authors:** Bhargav D. Mansuriya, Zeynep Altintas

**Affiliations:** Institute of Chemistry, Technical University of Berlin, Straße des 17. Juni 124, 10623 Berlin, Germany; b.mansuriya@campus.tu-berlin.de

**Keywords:** carbon dots, nanomaterials, electrochemical sensors, optical sensors, bioimaging, drug delivery, gene delivery, photodynamic therapy, photothermal therapy

## Abstract

Carbon dots (CDs) are usually smaller than 10 nm in size, and are meticulously formulated and recently introduced nanomaterials, among the other types of carbon-based nanomaterials. They have gained significant attention and an incredible interest in the field of nanotechnology and biomedical science, which is merely due to their considerable and exclusive attributes; including their enhanced electron transferability, photobleaching and photo-blinking effects, high photoluminescent quantum yield, fluorescence property, resistance to photo-decomposition, increased electrocatalytic activity, good aqueous solubility, excellent biocompatibility, long-term chemical stability, cost-effectiveness, negligible toxicity, and acquaintance of large effective surface area-to-volume ratio. CDs can be readily functionalized owing to the abundant functional groups on their surfaces, and they also exhibit remarkable sensing features such as specific, selective, and multiplex detectability. In addition, the physico-chemical characteristics of CDs can be easily tunable based on their intended usage or application. In this comprehensive review article, we mainly discuss the classification of CDs, their ideal properties, their general synthesis approaches, and primary characterization techniques. More importantly, we update the readers about the recent trends of CDs in health care applications (viz., their substantial and prominent role in the area of electrochemical and optical biosensing, bioimaging, drug/gene delivery, as well as in photodynamic/photothermal therapy).

## 1. Introduction

To date, a plethora of nanomaterials such as nano-magnetic beads [1], nanowires [2], nano-molecularly imprinted polymers (nano-MIPs) [3], polymer nanocomposites [4], dendrimers [5], metallic nanoparticles [6], carbon-based nanomaterials [7], and magnetic nanoparticles [8] are being exploited tremendously owing to their marvelous features, and they are efficiently applied in various fields, including photoelectric devices [9], micro-super capacitors [10], solar cells [11], optoelectronics [12], photodynamic therapy [13], photothermal therapy [14], electro- and photo-catalysis [15,16], environmental and food safety [17,18], novel drug delivery systems [19], new drug discovery [20], therapy development [21], theranostics and medical diagnostics [22,23], bioimaging [24], biosensing technology [25], etc. Amidst these nanomaterials, carbon-based nanomaterials are widely studied because of their indisputable prevalence in terms of biocompatibility, non-toxicity, inertness, eco-friendliness, long-term chemical stability, fluorescence properties, high electrical and thermal conductivity, large effective surface area, easy functionalization due to their abundant functional groups, excellent electro-catalytic activity, their ability to readily modify various electrodes during the construction of a wide range of biosensing platforms, etc. [26,27,28,29,30,31].

Carbon based nanomaterials usually encompass fullerenes (C_60_) [32], graphene (GR) [33], carbon black (CB) [34], carbon nanohorns (CNHs) [35], carbon nanodiamonds [36], carbon nanofibers (CNFs) [37], carbon nanotubes (CNTs) such as single-walled carbon nanotubes (SWCNTs) [38] and multi-walled carbon nanotubes (MWCNTs) [39], as well as quantum dots (QDs) such as carbon dots (CDs) [40]. All these carbon nanomaterials exhibit unique and unparalleled features that result in their huge exploitation for a variety of sensing applications such as disease diagnosis, metal ion detection, food and environmental regulations, etc. [41]. Additionally, some of these carbon nanomaterials are used as the fluorescent probes for bioimaging purposes. Moreover, they are also employed for certain therapeutic applications, where they can be served as nanocarriers for drug/gene delivery, besides acting as the therapeutic agents in photodynamic and photothermal therapies [42,43,44].

CDs are recently developed nanomaterials, among the other types of carbon-based nanomaterials. They are zero dimensional (0D) photoluminescent nanocarbon with usual size less than 10 nm [45,46]. Nonetheless, several reports have also showed that the CDs can also be significantly bigger in size, i.e., up to 60 nm [45]. The structure of CDs is comprised of sp^2^ and sp^3^ carbon atoms with large number of polymer chains or functional groups attached on their surfaces [47]. CDs have garnered enormous attraction among researchers, owing to their significant and outstanding characteristics, such as excellent electron conductivity, photobleaching and photo blinking properties, high photoluminescent quantum yield, fluorescence property, resistance to photo-decomposition, alterable excitation and emission attributes, increased electro-catalytic activity, good solubility in aqueous media, excellent biocompatibility, long-term chemical stability, cost-effectiveness, negligible toxicity, and acquaintance of large effective surface area-to-volume ratio [48,49,50,51]. CDs can be readily functionalized owing to the abundant functional groups present on their surface, and they also exhibit remarkable sensing features such as specific, selective, and multiplex detectability. The presence of abundant functional groups (e.g., amine, carboxyl, hydroxyl, etc.), or polymer chains on the surface of CDs results in an excellent solubility in aqueous solutions, as well as making them convenient for being functionalized with other nanomaterials [47,52,53]. The fundamental sp^2^/sp^3^ carbon skeleton of CDs usually displays amorphous carbon form or graphite lattice that can be due to the variation in their degree of carbonization [47,54]. In addition, the physico-chemical characteristics of CDs can be easily tunable based on their intended usage or application [54,55].

In the area of biomedicine and sensing technology, CDs are usually selected as the transducing elements or as the electrode modifiers, either in combination with other nanomaterials or as an individual nanomaterial for the desired sensing applications [56,57]. Additionally, they have an inimitable combination of electrical, mechanical, and optical characteristics to introduce miniaturized sensors with outstanding attributes for point-of-care-testing (POCT) [28,29,31]. CDs as the potent imaging agents play a pivotal role in the real-time imagining of either certain cells, tissues, organs, or in combination, that help in the accurate diagnosis of various forms of cancer and several diseases. Moreover, they have the tendency to efficiently deliver genes/drugs by serving as nanocarriers, as well as also being highly capable theranostic agents for various phototherapies like photodynamic and photothermal therapies [43,44,58,59,60,61,62,63]. Henceforth, all these significant attributes of CDs make them strong foundations for serving various diagnostic and therapeutic applications.

**Figure 1:**
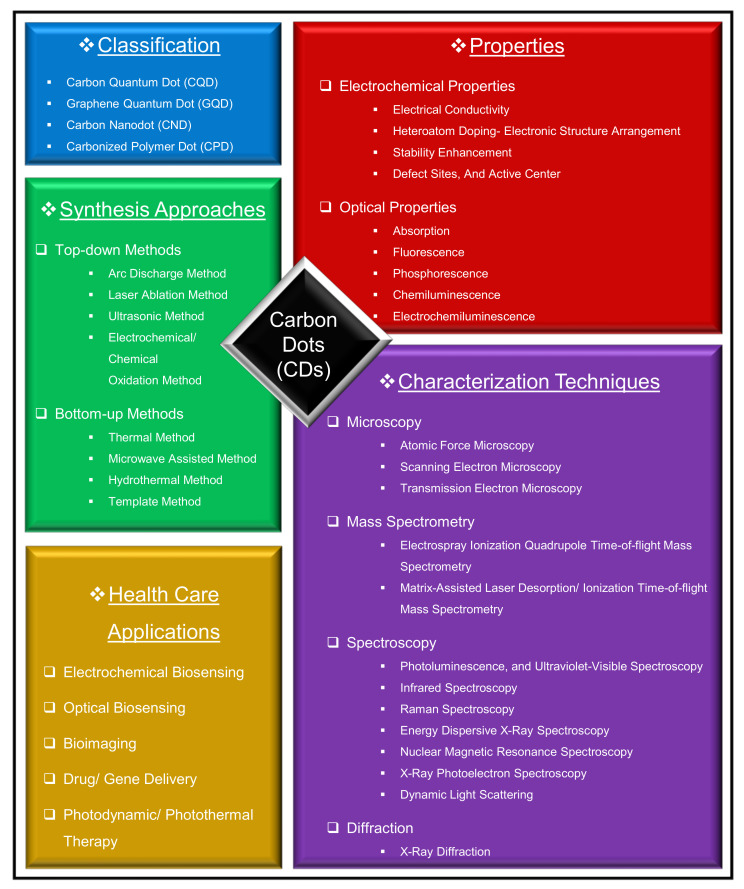
Overview of the classification of CDs, their properties, synthesis approaches, characterization techniques, and applications in health care.

In this comprehensive review article, we mainly discuss the classification of CDs, their ideal properties, their general synthesis approaches, and primary characterization techniques. More importantly, we update the readers regarding the recent trends of CDs in health care applications (viz., their substantial and prominent role in the areas of electrochemical and optical biosensing, bioimaging, drug delivery, gene delivery, as well as in photodynamic therapy, and photothermal therapy). In addition, we elaborate the fabrication steps, the operation details, as well as the performance characteristics of several recently reported electrochemical and optical sensors. Figure 1 outlines the list of topics that are covered in this comprehensive review article.

### Classification of CDs

**Figure 2:**
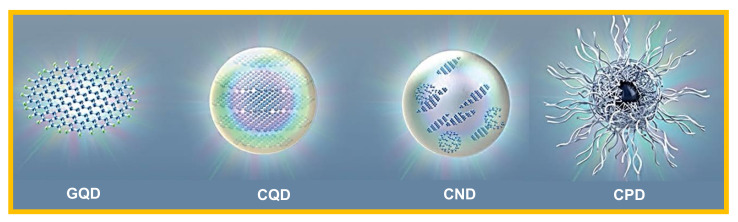
Classification of CDs. Adapted from [47], 2019, Wiley. GQD: graphene quantum dot; CQD: carbon quantum dot; CND: carbon nanodot; CPD: carbonized polymer dot.

The classification of CDs is based on their carbon core structure, surface functional groups, and their properties. As shown in Figure 2, CDs are classified into carbon quantum dots (CQDs), graphene quantum dots (GQDs), carbon nanodots (CNDs), and carbonized polymer dots (CPDs) [47,54].

The CQDs are nanospheres, crystalline in nature, and exhibit a large number of chemical groups that impart the intrinsic state luminescence and quantum confinement effect of CDs [53,64]. The GQDs are basically the tiny fragments of graphene, anisotropic in nature, and constitute mono- or multiple layers of graphene sheets with graphene networks in their configuration. GQDs have quantum confinement and edge effects because of the existence of various chemical functionalities on their edge or within their interlayer defect [25,51]. The CNDs possess a high degree of carbonization with edge effects, but without disclosing the crystalline or polymeric structures. Besides, CNDs lack in displaying the quantum confinement effect [65,66]. The CPDs are ideally the crosslinked nanohybrids of carbon and aggregated polymers, with a central carbonized core enveloped by either the polymeric chains or functional groups [67,68].

## 2. Properties of CDs

Owing to the outstanding properties offered by CDs, they are extensively employed in several biomedical areas, including biosensing, bioimaging, and therapy development. In this section, we elaborate the electrochemical and optical properties of CDs, which make them ideal candidates for health care applications, particularly in biosensing, bioimaging, drug/gene delivery, and photodynamic/photothermal therapy.

### 2.1. Electrochemical Properties of CDs

CDs are currently being engaged in the area of electrochemistry and electrocatalysis, by virtue of the following advantages.

(1) When compared to the other carbon-based nanomaterials, CDs exhibit exceptional charge transferability, enhanced electroconductivity, larger effective surface area, and lesser toxicity, as well as being comparatively cost-effective [48,49,50,51].
(2) The surface of CDs possesses abundant functional groups such as hydroxyl, carboxyl, amine, etc., which can deliver a large number of sites for the surface modification, as well as for the enhanced electrocatalytic activity by accelerating the intermolecular electroconductivity [53,69].
(3) When CDs are doped using heteroatoms such as nitrogen, phosphorous, sulfur, boron, etc., their electronic attributes can be significantly improved due to the intramolecular charge transferability [69,70,71].
(4) CDs can remarkably enhance electrocatalysis process during the electrochemical reactions such as oxygen evolution reaction (OER), hydrogen evolution reaction (HER), oxygen reduction reaction (ORR), and alcohol oxidation reaction (AOR) [69,72].

The above-mentioned merits of CDs make them ideal electrocatalytic agents to serve desired electrochemical applications.

#### 2.1.1. Electrical Conductivity

CDs display superior electrical conductivity, and when they are employed as electro-catalysts during the electrocatalytic reactions, the Schottky barrier occurring at an electrolyte-catalyst junction can be readily removed, which can thereby confirm the effective energy transformation [69,73]. Moreover, owing to the excellent electrical conductivity, CDs can very rapidly transfer electrons during electrochemical reactions [72].

#### 2.1.2. Heteroatom Doping- Electronic Structure Arrangement

When CDs are doped by heteroatoms such as nitrogen, phosphorous, sulfur, boron, etc., it results in the desired change in their chemical structure, whereas the electric charge can be efficiently transferred from the adjacent carbon atoms [71,74]. The heteroatom-doped CDs show exceptional electrochemical performance due to the enhancement of intrinsic activity of surface functional sites, the distortion of their electronic configuration, tuning of local densities, as well as the acceleration of adsorption and desorption phenomena [69,70,74].

#### 2.1.3. Stability Enrichment

Due to the presence of large numbers of active functional groups on the surfaces of CDs, as well as their long-term chemical stability in a wide range of solvents, they are proved to be the ideal nanomaterials in terms of improving the chemical stability of hybrid catalysts [69]. Furthermore, CDs can be used as the supporting materials during the preparation of their hybrid nanocomposites with metals and metal oxides, which can lead to the prevention of agglomeration and thereby, increase in the electrocatalytic activity [45,73]. In addition, CDs can demonstrate good stability in aqueous media due to the electrostatic stabilization, which can facilitate the steadiness of hybrid catalysts [53]. Moreover, due to the fact that CDs can strongly interact with catalysts via electrostatic interaction, they can therefore considerably enhance the stability [69,71].

#### 2.1.4. Defect Sites and Active Center

During electrochemical reactions, CDs can efficiently act as the active centers by virtue of their excellent electroconductivity, numerous defect sites and active edges, as well as their large surface area-to-volume ratio. Henceforth, when CDs are fused together with conductive materials, they can dramatically facilitate the electrochemical performance and characteristics [69,75,76].

### 2.2. Optical Properties of CDs

By virtue of the astonishing optical features offered by fluorescent CDs, they have been severely used in diverse health care applications, especially in the field of biosensing, bioimaging, and therapy development. It is of great importance to study and understand the optical properties of CDs in order to prepare a variety of CDs for serving multifarious bio-applications.

#### 2.2.1. Absorption Property

Due to the π–π* transition of C=C bonds in the structure of CDs, their absorbance is generated in the short-wavelength region. CDs reveal intense optical absorbance from 260 to 320 nm (i.e., in the UV region) [73]. Their absorbance range may vary depending on the type of CD, due to the surface functional groups, as well as their surface passivation [73,77].

#### 2.2.2. Fluorescence Properties

(a) Up-conversion fluorescence: It is the phenomenon, where the excitation wavelength is larger than the emission wavelength. The up-conversion fluorescence property can be observed in the CDs that are synthesized through ultrasonic treatment. Larger excitation wavelength results in the reduction of background autofluorescence, which is significant for the bioimaging application [53,77].
(b) Down-conversion fluorescence: The luminescent mechanism of CDs is yet to be deeply investigated. However, several origins responsible for the fluorescence of CDs usually include multi-emissive centers, free zigzag sites, self-trapped excitons, quantum confinement effects, special edge defects, their conjugated structures, and surface states [55,76,77,78]. Since CDs are 0D quantum confined nanomaterials, their fluorescence can be accredited to the presence of an electron-hole pair in their system [55]. As the size of CDs increases, their energy gap decreases. Therefore, the fluorescence property of CDs can be regulated by altering their quantum confinement effect [55,78,79]. The surface state phenomena due to the existence of surface functional groups and surface oxidation, is one of the other mechanisms for the origin of CDs’ fluorescence [76,80]. The surface oxidation incurred by oxygen-containing groups at the edge of CDs, is responsible for creating the surface defects that results in the fluorescence [76,78].
(c) Emission properties: Different fluorescence emissions of CDs can be obtained by controlling their excitation wavelength, which can be achieved by regulating several physicochemical parameters during CDs’ synthesis. For instance, the fluorescence of CDs is highly influenced by pH, concentration, as well as temperature [77]. The pH-dependent emission is because of the functional group protonation and deprotonation on their surfaces [81]; the concentration-dependent fluorescence is due to the surface state emission; whereas the temperature-dependent emission is the result of non-radiative decay occurring at the surface of CDs [77].
(d) Chemical stability and photobleaching properties: Fluorescence bioimaging or biosensing requires long emission lifetimes and stable fluorescence signal. This can be achieved with the help of CDs, since they have the tendency to produce stable signals when stored in an aqueous environment [82,83]. Furthermore, CDs can emit strong fluorescence for long time (i.e., up to a year). Generally, CDs are resistant to a broad pH range (i.e., from 3 to 12), therefore they demonstrate excellent impedance for photobleaching [81,83].

#### 2.2.3. Phosphorescence

The room-temperature phosphorescence (RTP) property of CDs is of great importance due to its long lifetime. Two aspects should be ideally taken into a consideration for obtaining RTP. The first involves the suppression of non-radiative transitions by restricting rotation and vibration, while the second one aims to facilitate the intersystem crossing ability by enriching the spin-orbit coupling through the use of transition metals [53,77]. Alternatively, RTP can also be achieved by employing CDs with enormously cross-linked structures containing non-conjugated groups [80]. The production of RTP in aqueous media is relatively challenging, since phosphorescence quenching is commonly observed in water because of the solvent-assisted relaxation, as well as due to the existence of dissolved oxygen [77,84].

#### 2.2.4. Chemiluminescence

In chemiluminescence (CL), the light is produced via a chemical reaction. Under appropriate conditions in redox reaction, CDs can generate CL in aqueous solvents, where the unstable products are produced from intermediate radicals during CL [53]. CDs can generate CL either due to their excitation after direct oxidation or through the enhancement or inhibition of their luminescence [77,82].

#### 2.2.5. Electrochemiluminescence

CDs are able to emit photons in the visible region under electrical excitation, which is important to study their electrochemiluminescence (ECL) property. Owing to the enhanced electron transfer due to a large amount of sp^2^ carbon in CDs, it results in a stable ECL [53,80].

## 3. Strategies for CDs Synthesis

The major factors influencing the synthesis of CDs include molecular state, surface state, as well as the quantum confinement effects, and these factors can be easily controlled by altering the strategies for CDs’ synthesis [85,86,87,88]. During the synthesis of CDs, several functional groups such as amine, epoxy, ether, carbonyl, carboxyl, hydroxyl, etc., can be incorporated [53,69]. Besides, the surface of CDs can be easily functionalized by doping them with heteroatoms like N, P, S, B, etc., using various biological, polymeric, and organic materials [71,74]. Henceforth, the properties of CDs can be regulated by varying the size and extent of the surface functional groups by either adapting different synthesis techniques or using other precursors [89]. The modification of CDs is crucial for achieving the considerable surface attributes for solvency as well as their favorable applications [53,76].

The quantum yield (QY) of CDs can be increased either while performing their syntheses or even after their preparation [90,91,92]. Over the last few years, significant investigations have been carried out in order to prepare CDs with high QY, for better bio-applications. Nonetheless, the high QY of CDs and their biocompatibility compete with each other, as well as it is quite difficult to regulate both of them individually. For the achievement of higher biocompatibility, surface passivation of CDs can be improved, which may result into the reduction in their photoluminescence intensity, and vice versa. Despite these issues, it is still unclear how CDs function as the excellent fluorophores for serving various health care applications [59,60].

Innumerable food-containing carbon sources can be employed to synthesize CDs with different QYs, e.g., yoghurt, honey, banana, pomegranate, leaves, sugar beet molasses, egg, rice bran, garlic, coffee beans, soybeans, coconut shell, tea leaves, grass, etc. [93,94,95,96,97,98,99,100,101]. Additionally, various green approaches for one-step synthesis of fluorescent CDs can be obtained using natural or synthetic non-toxic precursors for specific biosensing purposes, e.g., wool for synthesizing CDs to detect glycophosphate [102], sodium fluoride functionalized chitosan for preparing CDs to determine the retinoic acid content [103], etc.

**Figure 3:**
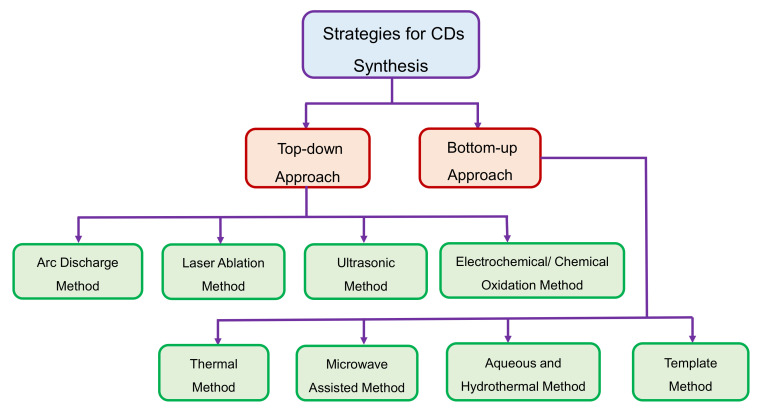
Approaches to synthesize CDs. Adapted from [49], 2018, Galenos Publishing House.

The synthesis of CDs can be achieved by one of the two strategies, i.e., either by top-down or by bottom-up approaches [104]. In the former case, carbonaceous materials are electrochemically, chemically, or physically dissected or cleaved into small nano-sized fragments [105]; whereas the latter approach involves either carbonization of small organic molecules or stepwise integration of small aromatic compounds [106]. As delineated in Figure 3, these two syntheses approaches of CDs are further classified into various methods [49,107].

### 3.1. Top-Down Approach

Currently, macroscopic carbonaceous materials including activated carbon, CNTs, and graphite are being extensively employed for the production of CDs by top-down approaches such as arc discharge method, laser ablation method, ultrasonic treatment, and electrochemical methods [45,80,108]. Nonetheless, these techniques are generally conducted under conditions such as high acidity, high potential, as well as high energy. Due to such harsh conditions, these top-down methods are relatively tedious, in comparison to the bottom-up approaches [109,110].

#### 3.1.1. Arc Discharge Method

The arc discharge method can be implemented to prepare CDs from crude CNTs, though CDs obtained via arc discharge treatment could exhibit low QYs [111]. In 2004, Xu et al. and Bottani et al. firstly produced CDs by arc discharge method using single walled CNTs and multi walled CNTs as carbon sources, respectively, through oxidation reaction [112,113]. Furthermore, Arora and Sharma reported that the arc discharge procedure can be employed to reorient the C-atoms, which are produced by disintegration of bulky C-precursors in order to attain high energy plasma within the reaction assembly during the synthesis of CDs [114]. In general, the arc discharge method involves abundant composite segments, where the purification of these segments is quite difficult [107].

#### 3.1.2. Laser Ablation Method

The laser ablation technique involves a high-energy laser pulse, where the bulky carbonaceous material is irradiated into a thermodynamic environment that produces high pressure and temperature. This results in increased heat, and the formation of plasma by evaporation. Subsequently, the generated vapor is converted into CDs by a crystallization process [49,104]. Through the laser ablation approach, Sun and the group revealed the use of argon as a carrier gas for water vapor along with carbon target to produce luminescent CDs [115]. In another study, fluorescent CDs with average size of 3 nm were synthesized via laser irradiation, which involved the suspension of carbon glassy particles in polyethylene glycol [116]. The as-synthesized CDs could be applied as fluorescent labels for in vivo bio-imaging of normal and cancerous human epithelial cells [116].

Organic solvents, such as amino-toluene, can be used to prepare nitrogen-doped CDs from graphite powder via a one-step laser ablation method [117]. With such an approach, CDs with excitation-independent wavelength can be produced for the determination of ratiometric pH, owing to the presence of abundant amine and oxygen groups on the surface of the CDs [117]. CDs obtained through double-beamed laser ablation could reveal superior features such as higher QY, ultra-small size (~1 nm), larger surface-to-volume ratio, better stability, and more homogeneity, when compared to those obtained by single-pulsed laser beam [118,119]. Therefore, double-pulsed laser ablation can be preferably applied to enhance the catalytic as well as sensing properties of CDs [119].

#### 3.1.3. Ultrasonic Method

In this method, ultrasound waves with low and high pressures generate small vacuum bubbles and distribute them uniformly throughout the solution [104]. As a consequence, it prevents aggregation, and results in the strong hydro-dynamic shear force and rapid infringement of the liquid jets [104,120]. By employing the energy generated via ultrasonic technique, large-sized carbon-based nanomaterials like graphite, activated carbon, and CNTs can be dissected into nanosized CDs [120,121].

Generally, the amine-functionalized CDs (NH_2_-CDs) are prepared by hydrothermal method involving harsh chemical reactions, several steps and high temperatures, which makes the synthesis of NH_2_-CDs quite expensive and tedious [122]. Therefore, using a facile approach, Wu et al. synthesized amine-functionalized CDs via ultrasonic method for cell imaging as well as for sensing nucleic acid, and metal ions like cobalt (II) ions [122]. In a study by Huang et al., one-step ultrasonic treatment of cigarette ash and thiol group-containing poly-ethylene glycol (SH-PEG) could yield PEG-decorated CDs with high QY for cell imaging application [123].

#### 3.1.4. Electrochemical/Chemical Oxidation Method

Due to the advantages offered by electrochemical/chemical oxidation, it is the most commonly used method for the preparation of CDs. This technique is very inexpensive, quick, and repeatable; and when applied, the size of CDs can be readily controlled, as well as obtaining highly pure CDs with high QY for bulk production [49,105,124]. Under normal pressure and temperature, the CDs are electrochemically or chemically synthesized via oxidation-reduction reactions [111]. It involves the use of strong oxidizing agents such as hydrogen peroxide (H_2_O_2_), sulfuric acid (H_2_SO_4_), nitric acid (HNO_3_), etc. [111]. The surface of CDs can be functionalized with hydrophilic groups such as ‒NH_2_, ‒COOH, ‒OH, etc., by regulating the redox reactions and the electrolyte components [111,125].

The electrolytes and the electrode materials are crucial, because they have the ability to produce CDs with distinct features in terms of their fluorescence emission, cytotoxicity, and surface states [126,127,128,129]. In this context, using graphite as an electrode material, Liu et al. proposed the facile synthesis of CDs for bioimaging application as well as for the detection of ferric ion (Fe^3+^) in water samples [128]. Herein, CDs with high crystallinity and size of ~4 nm were electrochemically synthesized via the oxidation of graphite in alkaline alcohols [128]. Through controlled chemical oxidation, strong oxidants like perchloric acid (HClO_4_) and HNO_3_ have the ability to incorporate C-atoms into small organic molecules and lead to their conversion into carbon containing materials as well as insertion into smaller sheets [104,130]. Considering that the microstructure of CDs affects their optical characteristics, Tan et al. prepared various microstructures of CDs for bioimaging purposes, where the graphitized activated carbon was selectively oxidized with oxidizing agents like HClO_4_ and HNO_3_ to obtain CDs with adjustable fluorescence [131].

### 3.2. Bottom-Up Approach

Bottom-up approaches are currently trending owing to their advantages such as promising practical applicability, involvement of non-toxic precursor molecules, cost effectiveness, easy instrumentation, and their precise control, as well as facile and convenient methodology [49,54,104].

#### 3.2.1. Thermal Method

Thermal decomposition is one of the most preferable methods for the synthesis of CDs, which involves the carbonization or pyrolysis of the large-sized carbonaceous precursors under higher temperatures [104,132]. The benefits of using this method reside in the mass production of CDs, cost-effectiveness, lesser reaction time, broader tolerance of precursors, solvent-free strategies, and easy synthesis [104,133]. Additionally, by using the thermal method, fluorescent properties of CDs can be optimized by controlling parameters such as reaction-mix pH, reflux duration, and reaction temperatures [104,134].

By using the thermal method, Shang and co-workers derived CDs by pyrolyzing citric acid as a precursor molecule, which was achieved by altering the carbonization degree [135]. In another study, Ma et al. proposed a strategy to develop large scale synthesis of N-doped CDs through one-step pyrolysis, where the obtained CDs could exhibit high fluorescence QY (~88%) and larger conversion rate (>80%) [136]. Wang and colleagues also procured CDs with high QY (~87%) by regulating their size during the synthesis procedure, which was achieved due to the oxygen-free property, and by directly carbonizing the carbon microcrystal precursors [137].

#### 3.2.2. Microwave-Assisted Method

Due to the fact that micro-waves contain a wide range of electromagnetic waves from 1 mm to 1 m, they offer accelerated energies for the decomposition of the chemical bonds that exist in precursor molecules [104,138]. The microwave-assisted method is relatively easy, highly affordable and quite rapid with less reaction times, and produces uniform heat for the homogeneous distribution of CDs [49,139].

Using the one-step microwave-assisted synthesis approach, Yu et al. produced CDs from two precursor molecules, i.e., triethylenediamine hexahydrate and phthalic acid. It was reported that this approach required only 1 min to synthesize CDs with wider emission wavelength, excellent biocompatibility, and strong green fluorescence [140]. Ghanem and team proposed a microwave assisted synthesis strategy for N-doped CDs, where 2,2-dimethyl-1,3-propanediamine and citric acid monohydrate were employed as nitrogen and carbon sources, respectively. In this method, the as-prepared N-doped CDs could display exceptional fluorescent features with higher QY [141]. In another study using microwave irradiation, fluorescent CDs were prepared using glucosamine onto a PEG @ chitosan co-polymer layer. The resultant CDs could show excellent chemical stability, good fluorescence intensity, and excitation-dependent fluorescence in biological matrices [142].

#### 3.2.3. Hydrothermal Method

Synthesis of CDs by hydrothermal carbonization (HTC) technique is non-toxic, environment-friendly, and inexpensive. It involves the reaction of organic solvents as the precursors and their sealing in a hydrothermal reactor under higher temperatures and pressures. Various raw materials, such as chitosan, citric acid, glucose, proteins, etc., can be used as precursors to synthesize CDs using the HTC technique [104,143,144,145,146].

Very recently, Hasan et al. prepared various forms of CDs using microcrystalline cellulose, furfural, and hydroxy-methyl furfural as precursors through HTC [147]. Under short-wavelength UV light, these CDs could exhibit green luminescence, and showed different absorption and emission properties, depending on the type of precursor employed [147]. Luo et al. used ethylenediamine, trisodium citrate dehydrate, and cysteine as precursor molecules for synthesizing CDs via the hydrothermal method, where the prepared-CDs could display bright blue emission, good biocompatibility, and excellent fluorescence stability [148]. Sun and colleagues proposed a hydrothermal approach for preparing nitrogen and sulfur co-doped CDs (N/S-CDs) from gardenia fruit as a source [149]. The resultant N/S-CDs were found to be ~2 nm in size and spherical in shape, and could revel excellent luminescence stability under UV light irradiation, at high salt concentrations and broad pH range [149]. In another study, hydrothermal synthesis of N/S-CDs was carried out by employing methyl blue, ethylenediamine, and citric acid as sources, where the prepared N/S-CDs could reveal excitation independent blue fluorescence emission [150].

#### 3.2.4. Template Method

Synthesis of CDs using the template method involves two steps, i.e., (i) calcination of the desired CDs in a suitable template or mesoporous silicon spheres, and (ii) etching step for the removal of supports [49,104]. However, this technique has not been used more frequently for CDs synthesis.

Using the template method, Kurdyukov et al. synthesized monodisperse spherical CDs with the size of ~3.3 nm and graphite-like structure. The strategy involved the introduction of organo-silane as precursor into pores of mesoporous silica particles, its thermal decomposition with CDs synthesis, and subsequent removal of the template [151]. Furthermore, Yang and co-workers reported a soft-hard template method to obtain CDs with alterable composition, sizes, photoluminescence characteristics, and crystalline degrees [152]. In this approach, ordered mesoporous silica (OMS) SBA-15 and copolymer Pluronic P123 were employed as the hard and soft templates, respectively. At the same time, four different organic molecules were used as the carbon sources that included phenanthroline (PHA), pyrene (PY), diamine-benzene (DAB), and 1,3,5-trimethylbenzene (TMB) [152].

## 4. Characterization Techniques for CDs

A wide range of characterization techniques are currently being employed to study the morphology (i.e., size, shape, and structure), topography, elemental composition, crystallographic information, size distribution, and granular orientation of various types of CDs prepared through different synthetic methods. These methods principally include the microscopy, spectrometry, spectroscopy, as well as the diffraction techniques.

### 4.1. Characterization of CDs by Microscopy

Various microscopic methods are most commonly employed for characterizing the morphology of CDs. Through these microscopic methods, direct determination of the particle size can be readily achieved by measuring the individual nanoparticles. Mostly, CDs are characterized by microscopies such as atomic force microscopy (AFM), transmission electron microscopy (TEM), and scanning electron microscopy (SEM).

#### 4.1.1. Atomic Force Microscopy

**Figure 4:**
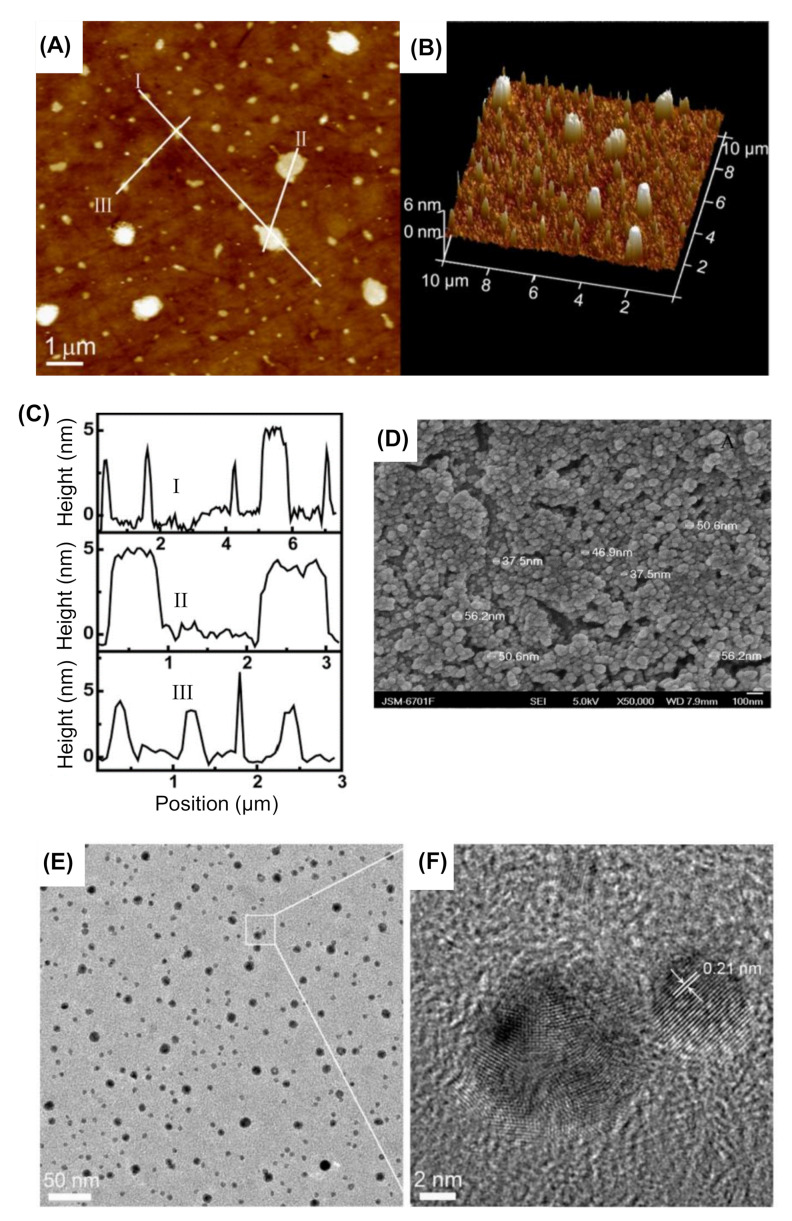
Illustrations of various microscopic images for the characterization of CDs. (**A**) 2D topographical AFM image. Reproduced from [156], with permission from Elsevier, 2014. (**B**) 3D topographical AFM image. Reproduced from [156], with permission from Elsevier, 2014. (**C**) The height profiles of lines I, II, and III that are mentioned in (A). Reproduced from [156], with permission from Elsevier, 2014. (**D**) SEM image. Reproduced from [157], with permission from Elsevier, 2014. (**E**) TEM image [158]. (**F**) HRTEM image [158].

AFM is a high-resolution scanning probe microscopic method, where a topographical image of the sample surface can be obtained depending on the interactions between a tip and a sample surface. A typical AFM is comprised of a cantilever with a small tip (probe) at the free end, a laser, a 4-quadrant photodiode, and a scanner. AFM facilitates the characterization of CDs by capturing their dimensional surface pictures at resolutions lower than 1 nm. When compared to other microscopies, AFM offers two-dimensional (2D) as well as three-dimensional (3D) information from the images of CDs. The dimensions of CDs can be measured via random computation of the particles’ heights on the 2D images, whereas 3D images determine the morphology of the surface of CDs [153,154,155]. The 2D and 3D topographical AFM images of CDs are illustrated in Figure 4A,B, respectively. Figure 4C shows the height profiles of lines I, II, and III that are mentioned in Figure 4A (i.e., 2D AFM images of CDs) [156].

#### 4.1.2. Transmission Electron Microscopy

TEM retrieves the chemical information and the images of CDs at a spatial resolution equal to the level of atomic dimensions. In TEM, the electron beam through which an incident light is transmitted via a thin foil specimen and transformed into elastically or inelastically scattered electrons, when the electron beam interacts with the sample of CDs. The ratio of the distance between the objective lens, the CD specimen, and the image plane are considered as magnified by the lens. Precise particle size of bright field images as well as dark field images are provided by the TEM, and it gives details about the morphology, composition, and crystallographic information of CDs as it utilizes energetic electrons [104,155,159]. Nowadays, high resolution TEM (HRTEM) is broadly employed for studying the lattice and surface imperfections of CDs. HRTEM generates an interference image by using scattered as well as transmitted beams [155]. The examples of TEM and HRTEM images of CDs are depicted in Figure 4E,F, respectively [158].

#### 4.1.3. Scanning Electron Microscopy

For the characterization of CDs, SEM is capable of capturing their images with spatial resolution. In SEM, the sample of CDs is exposed to the high-energy electron beam, where the resultant accumulation of charge produces an image that retrieves the information about their morphology, topography, chemical composition, granular orientation, crystallographic information, etc. [155,160,161]. The SEM image of CDs is exemplified in Figure 4D [157]. Both SEM and TEM can be employed to examine the presence or absence of CD aggregates, as well as to check the uniformity of the CD dispersion. When compared to SEM, the TEM has higher resolution power. Another disadvantage of using SEM resides in its limitation for imaging the large-scale of CDs. Therefore, TEM can be a favorable alternative to SEM, in cases where the measurement beats the SEM resolution [155].

### 4.2. Characterization of CDs by Mass Spectrometry

Mass spectroscopy is being employed as an eminent technique to characterize CDs, which enables elucidation of the chemical structures of desired nanosized CDs [155]. This method involves techniques such as electrospray ionization quadrupole time-of-flight tandem mass spectrometry (ESIQ-TOF-MS/MS) and matrix assisted laser desorption/ionization time-of-flight mass spectrometry (MALDI-TOF MS).

#### 4.2.1. Electrospray Ionization Quadrupole Time-Of-Flight Tandem Mass Spectrometry

**Figure 5:**
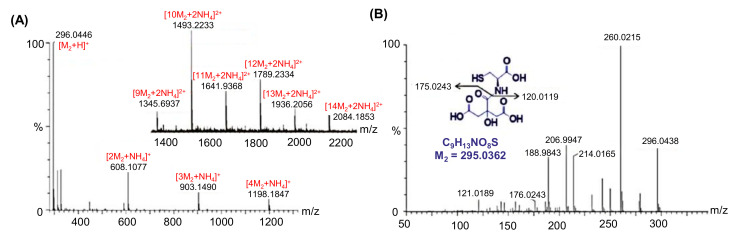
Representation of (**A**) MS spectrum. (**B**) MS/MS spectrum from ESI-TOF-MS analysis of CDs. Inset: MS spectra of the CDs in the higher m/z ranges [155].

ESIQ-TOF-MS/MS involves the use of sensitive MS detection as well as the soft ionization method [155]. By applying ESIQ-TOF-MS/MS, Hu et al. characterized the CDs through the combination of ESIQ-TOF-MS/MS with ultraperformance liquid chromatography (UPLC) [162,163]. As depicted in Figure 5, the MS/MS as well as the MS spectra were simultaneously captured for CDs’ characterization in order to gain the molecular formulae for individual CDs. These studies showed that CDs display supramolecular clusters with their respective monomer units coupled via noncovalent interaction [162,163].

#### 4.2.2. Matrix-Assisted Laser Desorption/Ionization Time-Of-Flight Mass Spectrometry

MALDI-TOF-MS is a soft ionization method, which has been employed to characterize the structure of CDs. In this method, mass of ions is determined by the difference in flight times, where the ions with the same energy levels are directed towards the detector. MALDI-TOF MS provides accurate mass as good as 0.1 [155,164,165].

**Figure 6:**
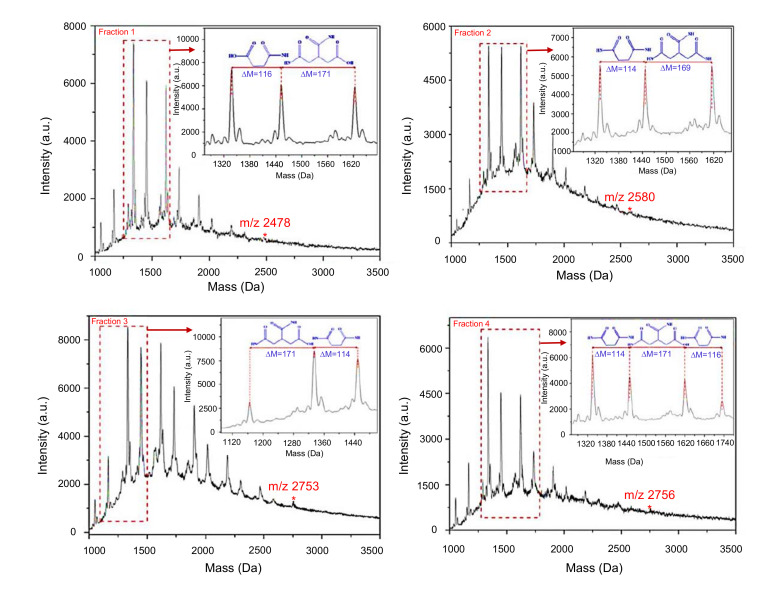
Graphical representation of different MS spectra from MALDI-TOF MS analysis of various fractions of CDs. Reproduced from [166], with permission from Wiley, 2014. a.u.: arbitrary units.

Using this technique, Hu et al. developed a strategy to characterize CDs depending upon their fragmentation [166]. The structural elucidation of CDs synthesized by microwave-assisted pyrolysis of 1,2-ethylenediamine and citric acid is described in Figure 6, which is a classic illustration of MALDI-TOF MS analysis [166]. In this study, the CDs were initially segregated using reverse phase high performance liquid chromatography (RP-HPLC), followed by the collection of fractions and further characterization using MALDI-TOF MS under a pulsed N_2_ laser in a positive ionization mode [166]. As a result, each fraction of CDs could exhibit the distinguished fragmentation pattern that was related to the amine/amide and carboxylic acid moieties attached on their surface [166]. Although this method is capable of recognizing the chemical functionalities of CDs, it is less sensitive for higher mass as well being unable to capture the bigger ions with higher mass range [155].

### 4.3. Characterization of CDs by Spectroscopy

Various spectroscopic techniques such as ultraviolet-visible (UV-Vis), photoluminescence (PL), infrared (IR), Raman spectroscopy (RS), energy dispersive X-ray (EDX), nuclear magnetic resonance (NMR), dynamic light scattering (DLS), and X-ray photoelectron spectroscopy have also been used for the characterization of CDs.

#### 4.3.1. Photoluminescence and Ultraviolet-Visible Spectroscopy

**Figure 7:**
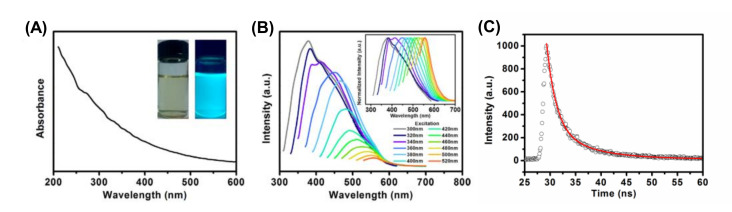
Characterization of S-CDs. (**A**) UV-Vis absorption spectrum. (**B**) PL spectrum at different λex. (**C**) Time-resolved PL. Reproduced from [169], with permission from Elsevier, 2014. a.u.: arbitrary units.

PL and UV-Vis spectroscopies are broadly employed methods for studying the optical characteristics of CDs. Additionally, these two spectroscopies can be particularly employed for calculating the QY of CDs [82,155,167].Usually, all forms of CDs show their activities in the UV-Vis region of the electromagnetic spectrum. Furthermore, fluorescence of CDs reveals λ_ex_-dependent emission. PL spectroscopy is most commonly used to determine the photoluminescent lifetime of CDs [82,155,167,168]. Figure 7 shows illustrations of UV-Vis absorption spectra, PL spectra at different λ_ex_, and time-resolved PL spectra of sulfur-doped carbon dots (S-CDs) [169].

#### 4.3.2. Infrared Spectroscopy

IR is a broadly employed technique for characterizing the chemical functionalities of CDs [170,171]. IR can not only evaluate the carbonyl (C=O) and hydroxyl (‒OH) groups on the surface of CDs, but it can also determine the heteroatoms doped on their surface [172,173,174]. Significant illustrations of IR analysis involving the recognition of the existence of boronic acid (B‒N and B‒O), phosphates (P=O and P‒OR), organosiloxane (Si‒OSi/Si‒O‒C), alkyl sulfide (C‒S), and amide/amine (‒CN/NH_2_) functionalities on the CDs’ surface provides confirmation about the incorporation of boron (B), phosphorus (P), silicon (Si), sulfur (S), and nitrogen (N), respectively [155]. The advantages of IR spectroscopy reside in easy sample preparation, rapidity, simplicity, and cost-effectiveness [155]. Nevertheless, IR is unable to retrieve the detailed information of CDs, e.g., about their doping with metal heteroatoms like nickel (Ni), magnesium (Mg), and aluminum (Al) [155]. Additionally, Fourier transform infrared (FTIR) spectroscopy can be used to characterize CDs containing hydroxyl, carboxyl, carboxylic acid, ether, or epoxy functionalities. FTIR is quite a robust method for analyzing these oxygen-rich functional groups [104].

#### 4.3.3. Raman Spectroscopy

**Figure 8:**
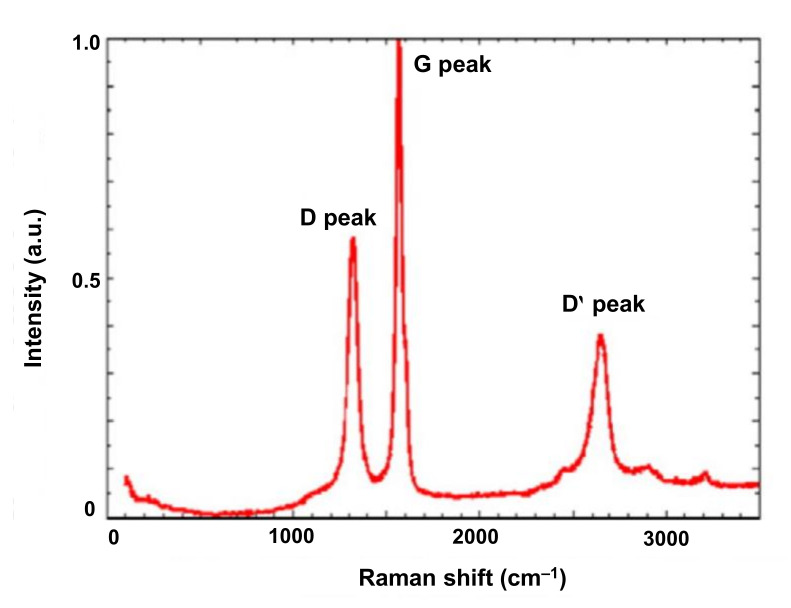
An illustration of Raman spectra of CDs. Reproduced from [181], with permission from Elsevier, 2013. a.u.: arbitrary units.

Raman spectroscopy is one of the most frequently applied non-invasive and non-destructive spectroscopic methods, which is employed for the determination of the carbon state in CDs samples [175,176,177]. Generally, Raman spectroscopy of CDs represents two major first order bands, i.e., G and D bands. The former is related to the vibration of sp^2^ C-atoms in a 2D hexagonal lattice, whereas the latter expresses the vibrations of C-atoms with dangling bonds in the termination plane of disordered glassy carbon or graphite. The intensity ratio (D/G) of these two bands determines the graphitization or degree of disorder of CDs that evaluates the purity of sample [155,178,179,180]. An example of Raman spectrum of CDs is shown in Figure 8, where both the G and D bands can be observed at 1578 cm^−1^ and 1331 cm^−1^, respectively [181]. The D/G ratio was calculated to be 0.59, which could signify the nanocrystalline graphite structure of CDs. The supplementary D′ band (2654 cm^−1^) represents the presence of sp^2^ hybridized C-atoms [181]. In some cases, it is difficult to obtain high resolution Raman spectra because of the strong fluorescent property of CDs, which hampers the generation of a clear Raman signal [155,182].

#### 4.3.4. Energy Dispersive X-ray Spectroscopy

**Figure 9:**
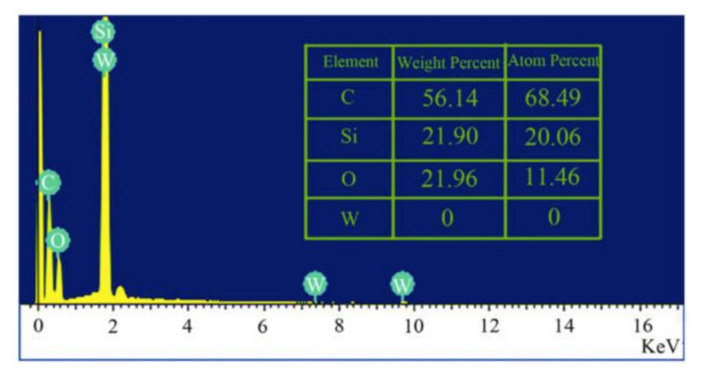
Representation of an EDX spectrum of CDs sample. Reproduced from [188], with permission from Elsevier, 2013.

EDS (also referred as EDX) is applied for analyzing the elemental composition of CDs, e.g., the content of C, N, and O in CDs, as well as the presence of other doped elements, such as silicon (Si). Additionally, this technique also provides information about the purity of CDs [183,184,185]. EDS involves the focusing of high-energy X-ray beams onto the CDs specimen to be evaluated. As a consequence of computing the energy and number of the X-rays radiated from CDs sample by employing energy dispersive spectrometer, an X-ray spectrum is generated [155,184,186]. Usually, quantitative and qualitative data of EDS spectrum are interpreted from the peak intensity and peak energy, respectively. These analyses are conducted based on the existing lines found in the EDS spectrum [155,187]. A typical example of an EDS spectrum for CDs’ characterization is depicted in Figure 9 [188].

#### 4.3.5. Nuclear Magnetic Resonance Spectroscopy

**Figure 10:**
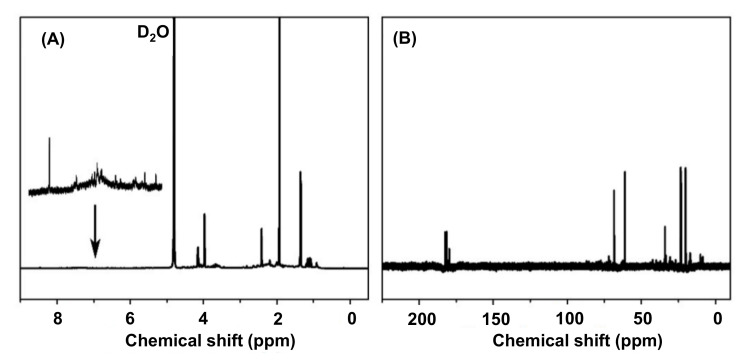
Characterization of CDs by NMR. (**A**) ^1^HNMR spectrum. (**B**) ^13^CNMR spectrum. Reproduced from [192], with permission from The Royal Society of Chemistry, 2017.

NMR spectroscopy is employed to provide additional and the important structural information for CDs. This technique enables recognizing of the chemical bond formations, elemental composition, as well as the functional groups present on the surface of CDs. Furthermore, NMR can identify the chemical modifications that occurred during carbonization due to the surface modifiers. Furthermore, using NMR for CDs’ characterization is quite easy, because of its non-destructive nature. Despite these advantages, NMR also exhibits considerable demerits such as lower sensitivity, greater time-consumption, and higher costs, when compared to the mass spectrometric methods [155,189,190,191]. Figure 10 embodies the illustrative NMR spectra for CDs’ characterization [192].

#### 4.3.6. Dynamic Light Scattering, and Zeta Potential Measurements

When coupled with liquid phase analysis armed with certain detecting systems, the DLS method is applied for the hydrodynamic particle size determination for CDs’ characterization. Through this technique, the radii of CDs can be evaluated via the measurement of diffusion rate of CDs in liquified media. However, the limitation of considering this method resides in unreliability and inaccuracy, because the DLS gives information only about the size distribution of CDs from the generated photon complementary function [155,193,194,195,196]. Furthermore, zeta-potential measurements can determine the surface charge as well as particle size of CDs, and can be broadly employed for the characterization of chemical functionalization on the surface of CDs [185,197]. The zeta-potential signifies the degree of repulsion between the similarly charged and adjacent particles in a dispersion, thereby regulating the stability of CDs. Additionally, this method provides information about the properties of CDs, such as double layer characteristics with various hydrophilic functional groups (hydroxyl, carboxyl, and carbonyl) [185,198,199].

#### 4.3.7. X-ray Photoelectron Spectroscopy

**Figure 11:**
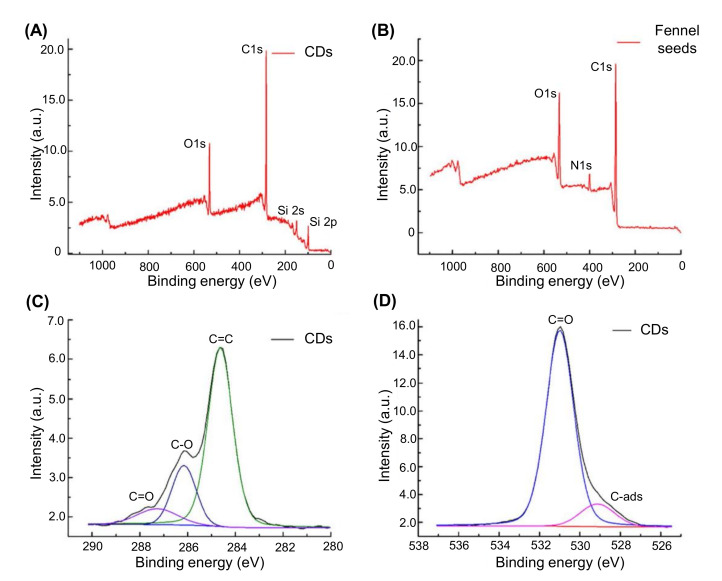
XPS spectra of: (**A**) CDs revealing O1s and C1s peaks. (**B**) Fennel seeds with C, N, and O composition. (**C**) C1s. (**D**) O1s [167].

XPS is used to characterize the electronic state of the elements present on the surface of CDs, their elemental composition, as well as their other surface attributes. In XPS, a beam of X-rays is directed at the CDs specimen, followed by the irradiation of the sample and concurrent measurement of kinetic energy and number of electrons [155,200,201,202]. Figure 11 demonstrates the ideal XPS spectra for CDs characterization [167]. Nonetheless, XPS is unable to determine individual nanoparticles, due to the lack of spatial resolution [155].

### 4.4. Characterization of CDs by Diffraction Technique

**Figure 12:**
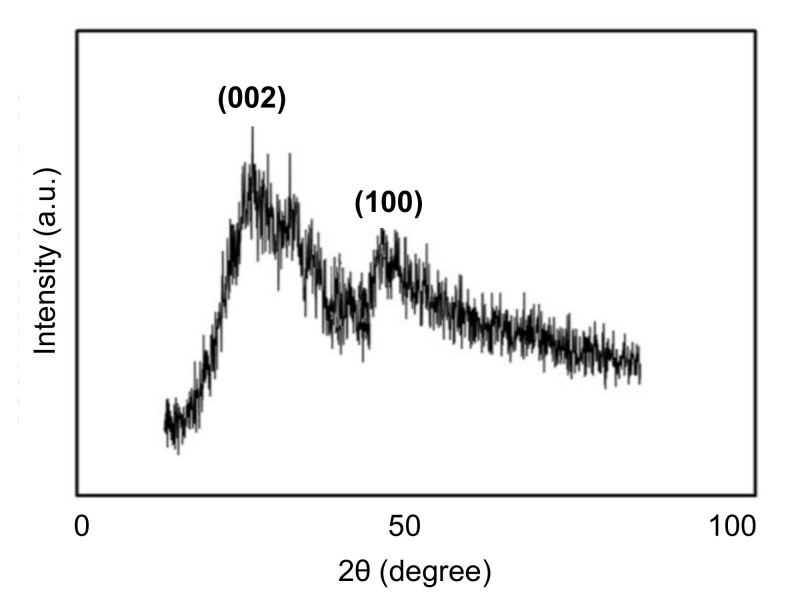
Representation of an XRD pattern for CDs. Reproduced from [173], with permission from Elsevier, 2019. a.u.: arbitrary units.

Characterization of CDs can also be achieved with the usage of X-ray diffraction (XRD) technique, from which the crystalline structure, phase purity, and particle size of CDs can be determined very quickly, depending on their diffraction pattern. When the X-rays are subjected to the crystalline structures of CDs, they are diffracted, detected, processed, and computed, which reveals the average structure of CDs. However, it is noteworthy to mention that XRD is inefficient to characterize amorphous CDs [155,194,201,203,204,205,206,207,208,209]. A classic example for the characterization of CDs by XRD is shown in Figure 12 [173].

## 5. Applications of CDs in Electrochemical Sensors

In the field of sensor technology, innumerable efforts were made over the past decade with an aim to produce reproducible, ultrasensitive, and innovative sensing platforms. These platforms may have the ability to specifically, selectively, and rapidly quantify diverse target analytes; ranging from various disease biomarkers, micro-organisms (e.g., fungi, viruses, bacteria, etc.), and metabolites, to heavy metal ions, toxins, pharmaceuticals, and glucose [48,210,211,212,213,214]. The identification of such analytes in their minute concentrations is very crucial for environmental and food safety; monitoring various diseases such as cardiovascular, neuro-degenerative, autoimmune, and infectious diseases; early diagnosis of cancer; as well as for new drug discovery [48,215,216,217,218]. To deliver this broad range of sensing applications, electrochemical sensors are extensively studied, and used as a miniaturized device for POCT in the area of health care and biomedicine [41,219,220].

Recent trends and enormous progress in the development of electrochemical sensors can be credited to the employment of variety of nanomaterials involved during their fabrication process [57]. Particularly, nanomaterials like CDs have shown significant contribution, and garnered tremendous interest in the construction of electrochemical sensors, owing to their attractive electrochemical properties mentioned in Section 2.1. In electrochemical sensors, signal transduction of biochemical reactions resulted in electrical responses by virtue of numerous electrochemical methods. These methods include voltammetry, amperometry, impedimetry, potentiometry, and conductometry. These sensors constitute a working electrode (W.E.) that serves as a transducer and recognition system, a counter electrode (C.E.), and a reference electrode (R.E.) for the measurement of electrical signal response [221,222,223,224,225]. These sensors are usually engineered by using one of the various types of receptors, which mainly include antibody, nucleic acid (DNA or RNA), aptamer, whole cell, molecularly imprinted polymer (MIP), enzyme, and peptide [25]. Nanomaterials, such as CDs, are preferred as transducing elements or as electrode modifier, either in combination with other nanomaterials or as an individual nanomaterial for the fabrication of desired electrochemical sensing platforms [57].

**Figure 13:**
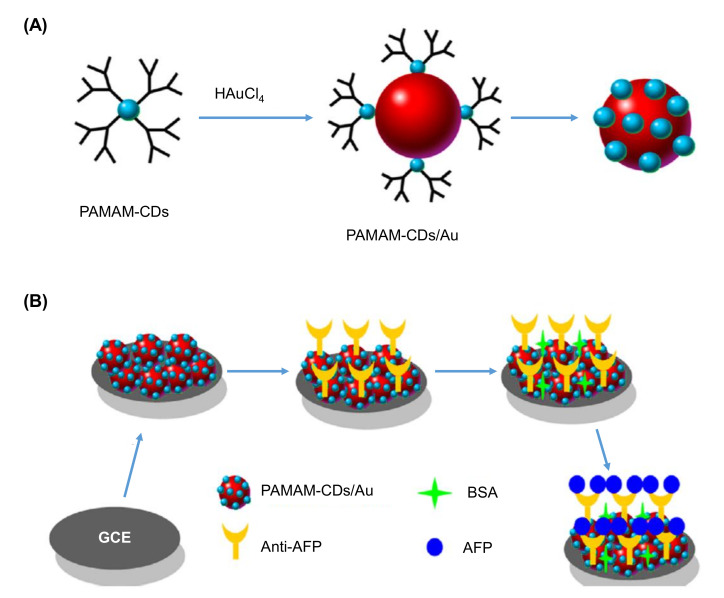
(**A**) Formation of PAMAM-CDs/Au hybrid nanomaterial. (**B**) Modification of GCE surface for the development of voltammetric immunosensor to detect AFP. Reproduced from [226], with permission from Elsevier, 2013.

Elevated levels of cancer biomarkers, such as α-fetoprotein (AFP), in human serum indicates early stage development of various cancer types such as epithelial ovarian tumors, nasopharyngeal cancer, and hepatic carcinoma [226]. Considering this, Gao et al. introduced a label-free electrochemical immunosensing strategy for the detection of AFP using a CDs-based antibody sensor, where the synthesis of CDs was accomplished using one-step microwave assisted pyrolysis of polyamidoamine (PAMAM) dendrimers and citric acid, with simultaneous surface passivation to obtain PAMAM capped-CDs [226]. As these PAMAM capped-CDs exhibit large numbers of ‒NH_2_ groups and can cause reduction of hydrogen tertachloroauric acid (HAuCl_4_), they were used as capping and reducing agents to produce PAMAM-CDs/Au hybrid nanomaterials. The use of CDs was intended for boosting the electroconductivity between the glassy carbon electrode (GCE) and the sensing interface. Furthermore, the PAMAM-CDs could increase the stability of PAMAM-CDs/Au on the GCE surface, owing to the thin layer formation by PAMAM dendrimer on the electrode surface as well as due to the electrostatic interaction. Uncoated Au nanoparticles on the GCE surface could provide abundant active sites and enhanced the effective surface area for anti-AFP immobilization. Prior to biomarker detection, the antibody-free areas on the as-fabricated GCE surface was covered using bovine serum albumin (BSA) [226]. The entire sensor fabrication process is depicted in Figure 13. Using differential pulse voltammetry (DPV), the anti-AFP/PAMAM-CDs/Au@GCE sensor could sensitively detect AFP antigen in a dynamic linear concentration, ranging from 1 × 10^2^ to 1 × 10^8^ fg mL^−1^, where the limit of detection (LOD) was estimated to be 25 fg mL^−1^. In this research work, PAMAM capped-CDs were characterized using UV-Vis spectroscopy, HRTEM, FTIR, and XPS techniques, whereas PAMAM-CDs/Au hybrid nanomaterials were characterized via SEM, TEM, HRTEM, XPS, and EDX. Moreover, the electrochemical techniques, such as DPV and electron impedance spectroscopy (EIS), as well as microscopy, such as SEM and TEM, could confirm the success of sensor fabrication [226].

CDs-based antibody sensors have also shown evidence in monitoring cardiovascular diseases like acute myocardial infarction (AMI). Very recently, Karaman and co-workers reported an electrochemical immunosensor for detecting heart-type fatty acid–binding protein (h-FABP), which is significant for the early diagnosis of AMI [227]. Herein, core-shell high-crystalline graphitic carbon nitride decorated carbon dots (hc-g-C_3_N_4_@CDs) and Cd_0.5_Zn_0.5_S/d-Ti_3_C_2_Tx MXene nanocomposite (MXene: Transition metal nitride or carbide) were employed as electrode modifiers for signal amplification. The hc-g-C_3_N_4_@CDs were prepared using the calcination method, followed by their implantation onto the surface of the GCE. Subsequently, the primary antibodies were bio-immobilized onto the surface of hc-g-C_3_N_4_@CDs. Thereafter, Cd_0.5_Zn_0.5_S/d-Ti_3_C_2_Tx MXene nanocomposites were fused with secondary antibodies via strong electrostatic and π–π interactions [227]. The characterization of as-constructed antibody sensors was achieved using EIS, cyclic voltammetry (CV), XPS, FTIR, XRD, SEM, and TEM. Using CV, DPV, and EIS techniques, this sandwich-type immunoassay could show a wide linear range from 0.01 to 1 pg mL^−1^ of h-FABP concentration, with an LOD of 3.3 fg mL^−1^ [227].

Using chitosan-CDs, Sarkar and team proposed an electrochemical immunosensing strategy for tracing the levels of vitamin D_2_ in milk samples [228]. Herein, the CDs were inhouse prepared using the microwave assisted technique, and their characterization was achieved by means of UV-Vis spectroscopy, FTIR, Raman spectroscopy, and TEM [228]. For the preparation of chitosan-CDs nanocomposite, CDs were gradually introduced into the solution containing chitosan. For sensor fabrication, indium tin oxide (ITO) glass substrate was laminated with a thin layer of chitosan-CDs via drop coating. Subsequently, the vitamin D_2_ antibodies were immobilized onto the surface of chitosan-CDs@ITO using EDC [(1-ethyl-3-(3-(dimethylamino)propyl) carbodiimide]- NHS (N-hydroxysuccinimide) coupling chemistry, followed by covering the antibody-free areas with BSA [228]. The as-modified ITO electrode was characterized by CV, static contact angle measurement, and AFM. DPV was employed to interpret the biochemical signal response generated for vitamin D_2_ antigen, where the sensor could detect vitamin D_2_ in a broad linear concentration, ranging from 10 to 50 ng mL^−1^, with an LOD of 1.35 ng mL^−1^ [228].

**Figure 14:**
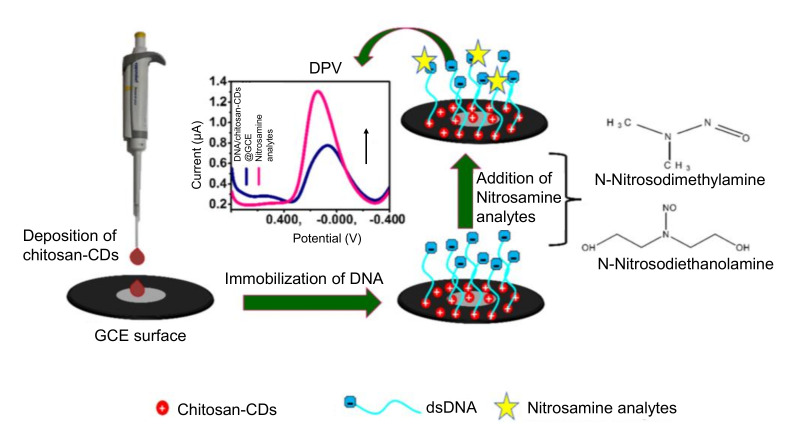
Steps involved in the construction of a chitosan-CDs based electrochemical DNA sensor for the detection of mutagenic nitrosamines. Reprinted from [212], with permission from American Chemical Society, 2020.

Majumdar et al. reported a CDs-based electrochemical DNA sensor to selectively and sensitively determine carcinogenic or mutagenic substances like mutagenic nitrosamines such as N-nitrosodiethanolamine (NDEA) and N-nitrosodimethylamine (NDMA) [212]. The sensor fabrication steps involved the deposition of chitosan-CDs on to the surface of GCE, followed by the electrostatic immobilization of DNA on chitosan-CDs surface (Figure 14). Using DPV, this sensor could quantify NDEA and NDMA in the wide linear concentration range from 9.6 × 10^−9^ to 4.02 × 10^−7^ M and 9.9 × 10^−9^ to 7.4 × 10^−7^ M for NDEA and NMDA, respectively, where the corresponding LOD values were estimated to be 9.6 × 10^−9^ M and 9.9 × 10^−9^ M [212].

An electrochemical DNA sensing strategy by Huang and the group was developed for the quantification of *colitoxin* DNA in human serum [229]. This research work involved the use of the one-pot microwave technique for the rapid and green synthesis of CDs with high QY, sourced from banana peels. Using a facile sequential reduction strategy, Pd-Au@CDs were prepared, where the CDs were employed as stabilizing as well as reducing agents. The GCE surface was then modified using these Pd-Au@CDs nanomaterials, followed by the immobilization of a single-stranded probe DNA via a carboxyl ammonia condensation reaction [229]. The nanomaterials were characterized using UV-Vis spectroscopy, XRD, XPS, HRTEM, EDX, IR, and Raman spectroscopy. CV and EIS methods revealed the accomplishment of sensor construction [229]. For the electrochemical detection of target DNA, the sensor surface could achieve a linear DPV signal response from 5 × 10^−16^ to 1 × 10^−10^ mol L^−1^, where the LOD value was found as 1.82 × 10^−17^ mol L^−1^. Moreover, this sensor has the ability to readily differentiate the base-mismatched, complementary, or non-complementary DNA sequences [229].

**Figure 15:**
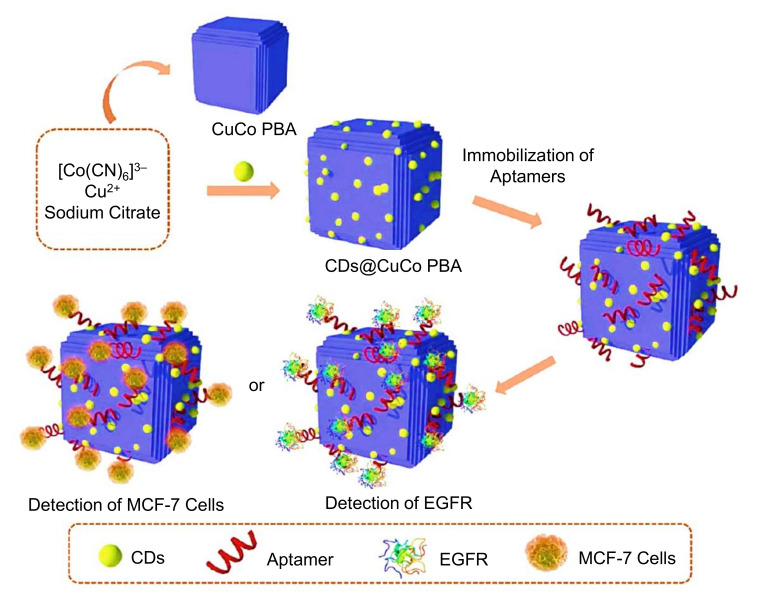
Graphical illustration of the fabrication of an aptamer-based biosensor, involving the use of CDs@CuCo PBA for the detection of EGFR, and EGFR-overexpressed MCF-7 breast cancer cells. Reproduced from [230], with permission from the Royal Society of Chemistry, 2020.

As displayed in Figure 15, Song et al. synthesized CDs coupled with bimetallic CuCo Prussian blue analogue (CD@CuCo PBA), and used it as gold electrode modifier to develop an electrochemical aptasensor for the detection of an epidermal growth factor receptor (EGFR) and EGFR-overexpressed MCF-7 breast cancer cells [230]. The CDs@CuCo PBA nanocomposite showed the ability to promote aptamer immobilization, and exhibited good biocompatibility, enhanced electrical conductivity, adjustable electronic configuration, active nitrogen species, high porosity, and large effective surface area [230]. FTIR, XRD, XPS, SEM, TEM, and HRTEM were employed for characterizing the CDs@CuCo PBA nanocomposite, while CV and EIS techniques were used to characterize the sensor fabrication. Using CV and EIS, this aptasensor could determine the LOD values for EGFR, and EGFR-overexpressed MCF-7 cancerous cells as 0.42 fg mL^−1^ and 80 cells mL^−1^, respectively [230].

Using molecular imprinting technology, Guo and co-workers constructed an MIP-based electrochemical sensor for the determination of patulin in fruit juice [231]. Herein, chitosan-CDs, 2-oxindole, and ρ-Aminothiophenol (ρ-ATP) were applied as GCE modifiers, pseudo template, and functional monomers, respectively. While fabricating the sensor, 2-oxindole and ρ-ATP were implanted onto the GCE surface via the hydrogen and Au–S bonds formation. Consequently, the polymeric solution constituted of the template molecule 2-oxindole, tetrabutylammonium perchlorate (TBAP), HAuCl_4_, and ρ-ATP was electropolymerized onto the GCE surface [231]. AFM, SEM, TEM, DPV, and CV were carried out to characterize the electropolymerized electrode surface. It was found that the DPV signal response was linearly increased over a dynamic range of patulin concentration from 1 × 10^−12^ to 1 × 10^−9^ mol L^−1^, where this MIP based-sensor could offer an LOD value of 7.57 × 10^−13^ mol L^−1^ [231].

**Figure 16:**
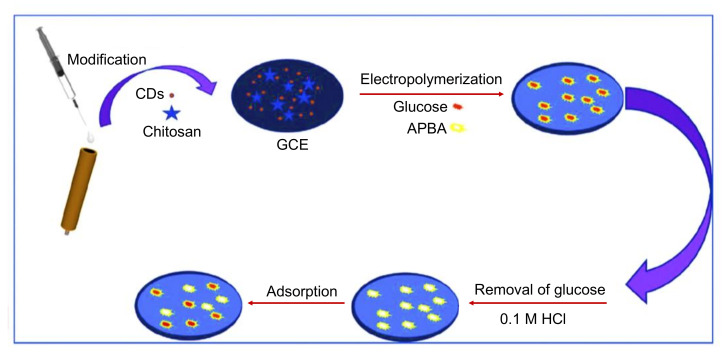
Fabrication steps of an MIP/chitosan-CDs@GCE surface. Reproduced from [232], with permission from Elsevier, 2018.

Zheng et al. established a strategy to develop a GCE-modified electrochemical MIP sensor for glucose detection [232]. As shown in Figure 16, chitosan-CDs, 3-aminobenzeneboronic acid (APBA), and glucose were employed as electrode modifier, functional monomer, and template, respectively. The sensor fabrication steps were evaluated using CV, EIS, EDX, FTIR, and TEM. Using CV and DPV, this MIP/chitosan-CDs@GCE modified sensor could detect glucose over a wide concentration range from 0.5 to 40 µM and 50 to 600 µM, with an LOD of 0.09 µM [232].

In 2019, a fluorine and nitrogen co-doped CDs (F,N-CDs) decorated enzyme-based amperometric biosensor was reported for the selective quantification of catechol in water samples [233]. The synthesis of F,N-CDs was carried out using the hydrothermal technique, where p-phenylenediamine and 5-fluorouracil were used as precursors. For the sensor development, F,N-CDs were grown on the surface of GCE, followed by the bio-immobilization of an enzyme called laccase (Lac). The F,N-CDs were characterized by TEM, HRTEM, NMR, FTIR, XPS, photoluminescence, UV-Vis, and Raman spectroscopy; whereas the cyclic voltammograms confirmed the successful formation of Lac/F,N-CDs@GCE [233]. Amperometric studies revealed that this Lac/F,N-CDs@GCE based enzymatic sensor could display much relatively lower LOD (i.e., 14 nM) than the conventional bioassay techniques for catechol determination. Furthermore, the authors claim that this sensor can be reused about 50 times under controlled conditions [233].

In recent years, CDs are also employed for the selective and specific electrochemical biosensing of a wide range of pharmaceuticals. For instance, a voltammetric CDs@CuFe_2_O_4_ functionalized biosensor was engineered for simultaneously quantifying isoniazid and rifampicin [234], nafion-assisted N-CDs/Cu_2_O nanocomposite modified electrochemical sensing platform for chlorpromazine detection [235], chitosan-CDs and hexadecyltrimethylammonium bromide coated GCE-based amperometric sensing strategy for mesalazine identification [236], and N-CDs laminated GCE-based amperometric and voltammetric device for sensing paracetamol and H_2_O_2_ [237]. Additionally, some noteworthy characteristics of several other CDs-based electrochemical sensors are listed in Table 1.

## 6. Applications of CDs in Optical Sensors

Optical biosensing platforms refer to one of the most commonly reported forms of biosensors. They are highly meritorious, when compared to the traditional methods of analysis, since they offer inexpensive, sensitive, specific, label-free, real-time, and direct detection of a wide range of analytes. Over the past decade, the research and development in the area of optical biosensing has been highly conducted across the world. Introduction of novel optical biosensing devices are implemented by coupling highly multidisciplinary concepts as well as multiple advanced approaches, such as chemistry, biotechnology, molecular biology, micro/nano-technology, microelectromechanical systems (MEMSs), and microelectronics [246,247,248,249,250,251].

An optical biosensor is a tool that is constituted of an emitting light source, a bio-receptor, a modulating element, and an optical detector for the interpretation of the optical response [251,252]. Optical sensing is achieved, when the optical field is exposed to be interacted with a biorecognition element. These sensors work under the principle of either surface plasmon resonance (SPR), optical wavelength-modulated spectroscopy, Förster resonance energy transfer or fluorescence resonance energy transfer (FRET), electrochemiluminescence (ECL), chemiluminescence (CL), photoluminescence (PL), or fluorescence [251,252,253,254,255,256,257]. To the very best of our knowledge from the recently reported research works, the majority of the CDs-based optical sensors work under the principle of fluorescence. In general, CDs are largely explored for the development of optical sensors, because of their unique optical properties that are previously mentioned in the Section 2.2.

**Figure 17:**
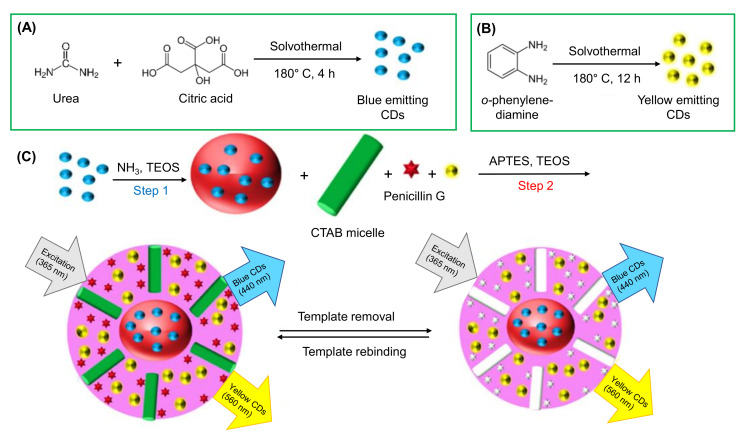
(**A**) Synthesis of blue emitting CDs. (**B**) Synthesis of yellow emitting CDs. (**C**) Schematic representation of a blue and yellow emitting CDs-based fluorescence MIP sensor for penicillin G detection. Reproduced from [258], with permission from Elsevier, 2020. TEOS: tetraethoxysilane; APTES: 3-aminopropyltriethoxysilane; CTAB: cetyltrimethylammonium bromide.

Jalili et al. engineered a blue and yellow emissive CDs-based fluorescence MIP sensor for the detection of antibiotics like penicillin G in milk samples [258]. In this ratiometric fluorescence sensing approach, these blue and yellow CDs, and a mesoporous structured MIP, served as a dual fluorophore and bioreceptor, respectively. During the detection of penicillin G, the quenching of fluorescence of the yellow emitting CDs was resulted, whereas the fluorescence of the blue emitting CDs remained constant. As a consequence, the color changed from yellow to blue [258]. Fluorescence spectra showed linear rise in the penicillin G concentrations, ranging from 1 to 32 nM, where the sensor could achieve an LOD of 0.34 nM in milk samples. The syntheses of blue and yellow emissive CDs as well as the sensor fabrication steps of this fluorescence-based penicillin G sensor is graphically described in Figure 17 [258].

In a study by Othman and colleagues, N-CDs were hydrothermally prepared from citric acid and urea, and employed as fluorescence labels for the development of an environment-friendly fluorescence-based immunosensor to rapidly and selectively identify nuclear matrix protein 22 (NMP22 antigen) in human urine samples [259]. Herein, the labeling of monoclonal antibodies with N-CDs was performed using EDC-NHS coupling chemistry to enhance the fluorescence intensity of N-CDs, followed by their incubation with a small concentration of NMP22 antigen for carrying out an immunoreaction between antibody and antigen [259]. Upon gradual addition of NMP22 antigen to the N-CDs/monoclonal antibody complex, the fluorescence intensity of N-CDs was found to be decreasing and it was quenched. Through this immunoassay, the detection of NMP22 was achieved in a broad linear concentration, ranging from 1.3 to 16.3 ng mL^−1^, with an estimated LOD value of 47 pg mL^−1^ [259].

**Figure 18:**
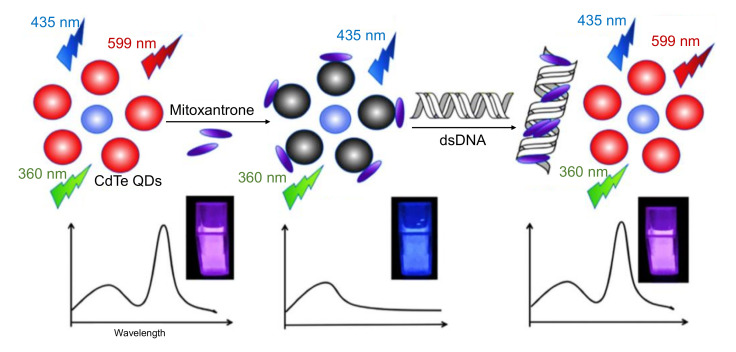
An example of fluorescence-based dsDNA detection method, using CdTe QDs/CDs/mitoxantrone complex. Reproduced from [260], with permission from Elsevier, 2017.

Using red emissive 3-mercapropionic acid-laminated cadmium telluride quantum dots (CdTe QDs) and green emissive water soluble fluorescent CDs, Liang et al. demonstrated a ratiometric fluorescence sensing strategy for double stranded DNA (dsDNA) recognition [260]. Under single-wavelength excitation of 360 nm, the emission peaks of CdTe QDs and CDs were observed at 599 and 435 nm, respectively. This sensor could detect target DNA by quenching the fluorescence intensity of CdTe QDs with the use of mitoxantrone. In the existence of DNA, the quenched fluorescence intensity of CdTe QDs was restored (Figure 18). On the contrary, the fluorescence intensity of CDs was constant. This fluorescence-based DNA sensor could achieve an LOD of 1 nM, and displayed a linear correlation over the concentration range of 0–50 nM for the target dsDNA [260].

Liao and co-workers carried out hydrothermal synthesis of N-CDs from urea and cellobiose for the construction of a label-free ratiometric fluorescence sensor to determine phosalone, an organophosphorus pesticide [261]. Only one emission peak at 420 nm resulted upon excitation at wavelengths of 327 or 235 nm. When the phosalone was introduced, the quenching of N-CDs was achieved at 235 nm within just 1 min, whereas the excitation peak at 327 nm displayed a negligible change. Correspondingly, the fluorescence intensity of N-CDs-phosalone complex was quenched at 254 nm, but it could not quench the fluorescence intensity of CDs at 365 nm [261]. Under controlled parameters, this N-CDs-based fluorescence sensing system could display a linearity for two different concentration ranges of phosalone, i.e., from 0.08 to 4 µg mL^−1^, and from 4 to 14 µg mL^−1^. Moreover, this sensor could demonstrate the phosalone detection with an estimated LOD value of 28.5 µg mL^−1^ [261].

Using fluorescent N-CDs, Kong et al. established an ultrasensitive, easy, and quick sensing tool to trace the levels of ascorbic acid in human serum samples, where the preparation of highly water soluble and biocompatible N-CDs was carried out using the hydrothermal method [262]. For the synthesis of N-CDs, tripolycyanamide and sodium citrate were used as nitrogen and carbon sources, respectively. Due to the additive combined influences of static quenching effect (SQE) and inner filter effect (IFE), the fluorescence intensity of N-CDs was linearly decreased with gradual introduction of ascorbic acid. This can be attributed to the formation of hydrogen bonds between the surface functionalities of N-CDs and the hydroxyl groups of ascorbic acid [262]. Authors claimed that this N-CDs-based sensor could sense ascorbic acid in a wide concentration range of 10^−3^−10^−8^ M within just 2 min, with the computed LOD value being 5 nM [262].

**Figure 19:**
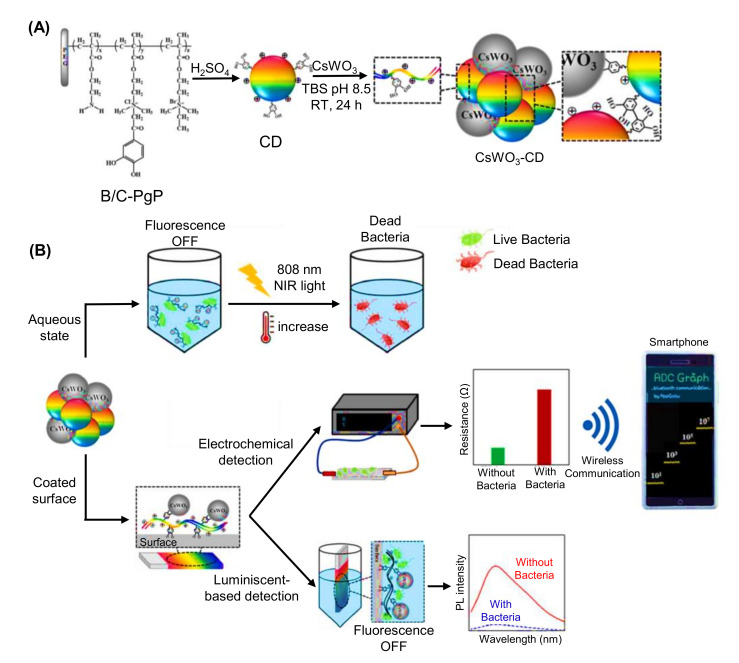
(**A**) Preparation of CsWO3-CD nanohybrids. (**B**) Scheme of luminescent-based and electrochemical sensor involving the use of CsWO_3_-CD nanohybrids to detect bacteria. Reproduced from [263], with permission from Elsevier, 2020. B/C-PgP: bromoethane- and 2-chloro-3,4-dihydroxyacetophenone- quaternized poly ethylene glycol-grafted poly-2-(dimethylamino)ethyl methacrylate; TBS: Tris- buffered saline; RT: room temperature; NIR: near infrared.

Robby and team reported a novel, versatile, sensitive, and effective wireless luminescent-based as well as electrochemical biosensing approach for simultaneously killing and detecting bacteria [263]. In this approach, as portrayed in Figure 19, the cesium tungsten oxide (CsWO_3_) coupled catechol-fused, cationic fluorescent CDs were employed for the assistance of photothermal-dependent antibacterial activity. The as-generated CsWO_3_-CD nanocomposites could demonstrate a strong fluorescence emission that could result in a quenching because of interacting the anionic bacterial cell wall with cationic CDs. As a consequence, a significant difference in the resistance values was observed upon the bacterial binding [263]. Herein, *Staphylococcus aureus* (*S. aureus*) and *Escherichia coli* (*E. coli*) were selected as the model bacteria. By applying the electrochemical technique for bacterial detection, the sensor could display an LOD value of <10 cfu mL^−1^ for both *S. aureus* as well as *E. coli*. At the same time, upon the application of the luminescent-based technique, the LOD values were found to be 131 cfu mL^−1^ and 70 cfu mL^−1^ for *S. aureus* and *E. coli*, respectively. This implies that the luminescent approach was less sensitive than the electrochemical method [263]. In addition, these CsWO_3_-CD nanocomposites could successfully kill all of the *S. aureus* and *E. coli* bacteria with just 1 mg mL^−1^ concentration of CsWO_3_-CDs. This could be attributed to the photo-thermolytic killing of bacteria, which was resulted due to the photothermal conversion of CsWO_3_, upon exposure to NIR light [263].

An antibody-independent fluorescence-based triple-module bioassay for sensing *Helicobacter pylori* (*H. pylori*) in human feces was designed using CDs [264]. The very first module (module I) constituted the *H. pylori* specific aptamer coupled with the Ca^2+^-functionalized superparamagnetic nanomaterials for the selective detection of *H. pylori* in human stool. The second module (module II) consisted of a bifunctional co-polymer of chloroprotoporphyrin IX iron (III)- polyethylene glycol-desferrioxamine in order to achieve high affinity binding with *H. pylori*, whereas the third module (module III) was comprised of Fe^3+^-quenched CDs [264]. Upon the interaction of module I/module II conjugate with module III, the scavenging of Fe^3+^ was achieved via magnetic separation, which could cause the transduction due to the restoration of quenched fluorescence of CDs. With this bioassay, *H. pylori* could be quantified in human feces samples over a dynamic concentration, ranging from 10 to 10^7^ cfu mL^−1^, with the detection limit of 1 cfu mL^−1^ [264].

**Figure 20:**
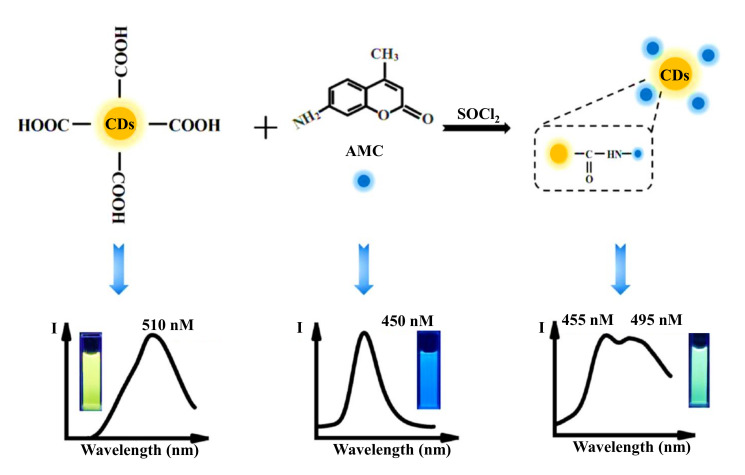
A graphical illustration of a dual-emission ratiometric fluorescence sensing method using 7-amino-4-methylcoumarin (AMC) and carboxylated-CDs nanohybrid. Reproduced from [265], with permission from Elsevier, 2020.

A versatile dual-emission ratiometric fluorescence sensor was constructed using amide-linked 7-amino-4-methylcoumarin (AMC) and carboxylated-CDs nanohybrid for the rapid determination of dopamine [265]. As shown in Figure 20, under a single excitation wavelength of 300 nm, two emissions were generated at 495 nm and 455 nm by AMC and CDs, respectively. The dopamine could quench the fluorescence of CDs, where the detection of dopamine was investigated on the basis of change in the ratiometric ratio of the fluorescence intensity. This sensor could display a linearity for a wide range of dopamine concentrations, ranging from 0 to 33.6 µM, with an LOD value as low as 5.67 nM [265].

A novel CDs and ferrocene-based fluorescence sensing strategy was implemented for selectively and sensitively determining peroxynitrite [266]. This strategy was designed on the basis of covalent conjugation of ferrocene carboxylic acid with CDs, and it could efficiently work under the principle of photoinduced electron transfer (PET) from ferrocene to CDs. It was reported that, when the peroxynitrite was introduced, the energy levels of the highest occupied molecular orbital (HOMO) of CDs were found to be suppressed because of the nitration and oxidation abilities. Henceforth, the fluorescence intensity of CDs could be quenched due to an effective electron transfer from ferrocene to the excited states of CDs [266]. This sensor could efficiently sense peroxynitrite with the detection limit as low as 2.9 nm, with wide linear concentrations of peroxynitrite, ranging from 4 to 120 nM [266].

Using S-nitrosothiol and green emissive CDs, Yue et al. reported a fluorescence sensing method for nitrite detection in food samples, where the detection mechanism was based on the principle of inner-filter effect between S-nitrosothiol and CDs [267]. In this study, the generation of S-nitrosothiol resulting via π-conjugation induced nitrite-thiol reaction, while the preparation of green emissive CDs was performed using hydrothermal technique, and were employed as the fluorescent probes. This sensing method could determine the nitrite concentrations in a broad linear range from 0.4 to 20 μg mL^−1^, with an estimated LOD value of 0.23 μg mL^−1^ [267].

**Figure 21:**
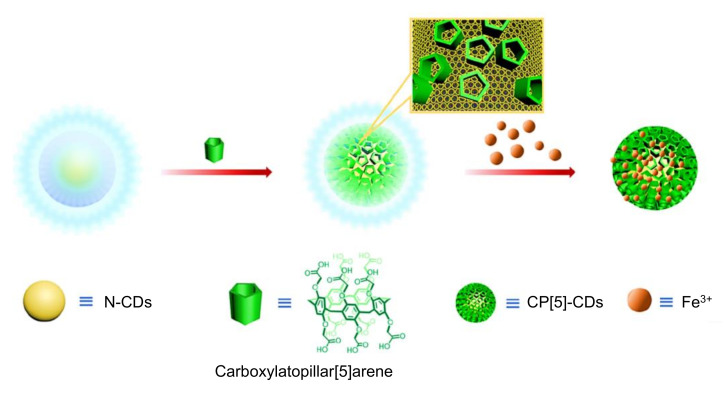
Schematic representation of Fe^3+^ detection using CP[5]-CDs [268].

Figure 21 depicts the functionalization of N-CDs with carboxylatopillar[5]arene (CP[5]) via covalent interaction to obtain CP[5]-CDs for the development of a fluorescence sensor for Fe^3+^ ions detection [268]. Upon the addition of Fe^3+^ ions to CP[5]-CDs, the Fe^3+^ ions resulted into the reduction in the fluorescence intensity of CP[5]-CDs nanohybrid, thereby led to the quenching of fluorescence. The fluorescence spectra displayed a linear calibration plot from 0 to 190 µM of Fe^3+^ concentration, and the sensor could demonstrate an LOD value of 1.2 µM [268]. Additionally, Table 2 lists a few important features of various other CDs-based fluorescence sensors.

In addition to the above-discussed CDs-based sensors, they are also being studied for the development of gas sensors. Recently, several gas sensors involving the use of CDs were reported. For instance, El-Shamy designed a CDs-functionalized magnesium oxide (MgO) nano-particle (CDs@MgO) sensor for sensing hydrogen sulfide (H_2_S) gas [282], Ramos-Ramón et al. developed a N-CDs based sensor for CO_2_ [283], Wang et al. fabricated silica aerosol-modified CDs-based gas sensor for NO_2_ monitoring [284], and Carbone prepared a CDs-assembled nickel oxide (NiO)-based sensor for methane gas detection [285]. However, most of the CDs-based gas sensors reported to date were developed for environmental monitoring and safety, it is speculated that CDs-based gas sensors will be a promising research area in the near future for serving biomedical applications as well.

## 7. Applications of CDs in Bioimaging

Bioimaging entails several techniques, which include positron emission tomography (PET) ultrasonic imaging, computed tomography imaging (CT), magnetic resonance imaging (MRI), optical imaging, etc. [59]. These techniques deliver images of biological materials, as well as their spatial and molecular details based on the type of required bioimaging application. Amidst these methods, fluorescent imaging has attained significant attention, because it is facile, affordable, and highly sensitive, as well as it generates high resolution photography [44,59,60,61,63].

CDs acquire immune system evasion, easy clearance from the body, resistance to photobleaching and swelling, weak interactions with proteins, excellent permeability and biocompatibility, multi-color emission profile, and low toxicity. These characteristics make them highly special candidates to serve as fluorescent probes for fluorescence labeling and bioimaging applications, in order to introduce novel bio-diagnostic approaches [43,60,63,286,287]. CDs can achieve diagnosis of various types of cancer, as well as other diseases, due to their capability to provide real time 3D photography and some significant information about the location, size, and type of tumors in the human body. The difference in the structures and shapes of different cells or tissues have a particular response to the foreign substances like CDs. Therefore, the synthesis and functionalization of CDs are carried out, depending on the requirement of the specific bioimaging application [43,59,61,62,288,289].

**Figure 22:**
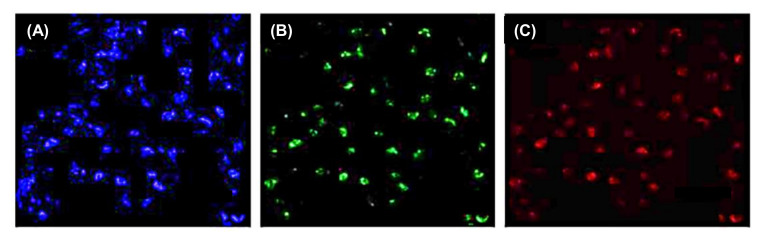
Bioimaging of HeLa cells. (**A**) Blue emission at 405 nm excitation. (**B**) Green emission at 458 nm excitation. (**C**) Red emission at 514 nm excitation [290].

By utilizing tetraphenylporphyrin or its transition metal (Pd or Pt) complex, Wu et al. synthesized nitrogen and metal co-doped CDs via the hydrothermal technique [290]. Herein, the as-prepared CDs were used as fluorescent probes for cellular multicolor imaging. Using MTT assay, the significance of low cytotoxic CQDs was examined for the bioimaging of HeLa cells. An investigational study was performed for confirming the usage of photoluminescent CDs in the bioimaging of living cells [290]. Figure 22 confirms the consistency of excitation-dependent photoluminescent feature of CDs, where the HeLa cells upon treatment with CDs, could generate blue, green, and, red emissions, under 405, 458, and 514 nm, respectively. It was found that most of the CDs were accumulated on the cellular cytoplasm rather than on the cellular nucleus. Henceforth, it implied the internalization of CDs into the cells via endocytosis [290].

Bhatt and the group prepared N-CDs from urea and ascorbic acid, and used them for bioimaging the Cu^2+^ and Hg^2+^ ions in the gastrointestinal track of *Artemia*, a brine shrimp [291]. This strategy was applied to evaluate the toxicity of *Artemia*, where the mortality was computed upon the exposure of N-CDs to *Artemia*. As a result, mortality rate was found to be only 5%, suggesting the non-toxicity of N-CDs. Upon excitation at 395–475 nm and 330–385 nm, *Artemia* could emit green and blue colored fluorescence, respectively. Henceforth, it can be inferred that N-CDs could be readily able to internalize the cell membrane with no surface passivation, and so, they can be applied as fluorescent probes for the bioimaging of *Artemia* [291]. Furthermore, considering the fact that *Artemia* exhibits the tendency to accumulate various heavy metal ions, N-CDs were further used to examine the presence of the Cu^2+^ and Hg^2+^ ions in *Artemia*. Upon incubation with *Artemia*, N-CDs displayed a strong fluorescence in the presence of Hg^2+^and Cu^2+^ ions [291].

**Figure 23:**
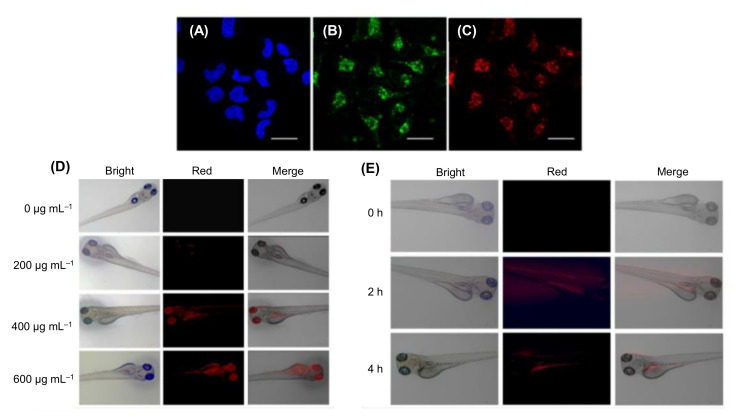
(**A**) Blue, (**B**) green, and (**C**) red emission of Ru-CDs for the bioimaging of HeLa cells. (**D**) Bioimaging results, and (**E**) fluorescence intensities of zebrafish embryos. Reprinted from [292], with permission from American Chemical Society, 2020.

Ruthenium-decorated CDs (Ru-CDs) from citric acid and 5-amino-1,10- phenanthroline ruthenium (II) complex were hydrothermally synthesized by Yue et al. for bioimaging HeLa cells and zebrafish embryos, owing to their strong luminescence property [292]. Figure 23A–C depicts the fluorescence emission of Ru-CDs for HeLa cells bioimaging, where the red emission (Figure 23C) was resulted in cytoplasm, which suggests that the CDs were accumulated in cancerous cells. For detailed investigation and bio-analysis, Hoechst 33342 and LysoTracker Green stainings were applied for differentiating the lysosomes and cellular nucleus, respectively. As portrayed in Figure 23A–C, the red fluorescence of Ru-CDs covered the blue emission, and protruded with that of the green fluorescence emission. This indicated that most of the Ru-CDs were present in the lysosomes of HeLa cells [292]. Moreover, due to the red fluorescence emission of Ru-CDs, they were also used as fluorescent probes for in vivo zebrafish embryo bioimaging (Figure 23D,E). With the increasing concentrations of Ru-CDs, the red fluorescence emission of zebrafish embryos was found to be more intense, implying the efficient accumulation of Ru-CDs in zebrafish embryos [292].

Due to the extremely low toxicity and excellent biocompatibility of CDs prepared from cumin as a carbon source, they have shown efficient bioimaging applications for human cancer cells, such as MCF-7 breast cancer cells, as well as for multi drug resistant (MDR) bacterial cells, such as *S. aureus* and *Pseudomonas aeruginosa* (*P. aeruginosa*) [293]. In the corresponding study, the *S. aureus, P. aeruginosa,* as well as the MCF-7 breast cancer cells were cultured for over 24 h, which was followed by the removal of culture media and addition of CDs. The exposure of these cells with CDs was allowed for a 24 h incubation, prior to capturing their respective fluorescence images [293]. Herein, the MMT assay was employed for determining the cytotoxicity of CDs for the MCF-7 breast cancer cells, where the results revealed 80% of cell viability, suggesting the feasibility of CDs for bioimaging. The fluorescence images could offer the information about the penetration of CDs into the cellular nucleus as well as cytoplasm of MCF-7 breast cancer cells, despite their major diffusion into the cell membrane [293]. Furthermore, when CDs were applied for bioimaging of the *S. aureus* and *P. aeruginosa* cells, very negligible values were found for the minimum inhibitory concentrations (MIC), which confirmed that the as-prepared CDs could neither show bactericidal nor bacteriostatic activity. Once having incubated the CDs with these *S. aureus* and *P. aeruginosa* cells, the fluorescence images displayed red, green, and blue emissions of CDs at the wavelengths of 545–570 nm, 450–490 nm, and 365–395 nm, respectively. These multi-color emissions revealed the successful incorporation of CDs into the *S. aureus* and *P. aeruginosa* cells [293].

## 8. Applications of CDs in Drug Delivery, and Gene Delivery

Using various fabrication and surface passivation techniques, the surface of CDs can be modified by functionalizing with polymeric materials, metallic nanoparticles, small organic molecules, etc., through ionic, hydrogen, or covalent bond formation, which may lead to the enhancement of photoluminescence, as well as water solubility features of CDs. These altered CDs can be efficiently employed for a wide range of theranostic applications, especially in drug delivery, and gene delivery. Moreover, CDs can be combined with genes or drugs for functioning as the targeted and imaging-regulated nanocomplexes to enhance the gene/drug delivery, and to develop the essential therapeutic approaches [43,59,60,294]. In this section, we very briefly discuss the potential role of CDs as the nanocarriers for drug or gene delivery by means of a few illustrations.

### 8.1. Role of CDs in Drug Delivery

The requirement of targeted drug delivery is to develop a new drug delivery system with a tendency to transport drug molecules to a specific target in the body that may result in an appropriate target-drug interaction. One of the most significant ways for improving the drug delivery system is to conjugate drugs with nanomaterials, which can result in the enhancement of drug absorption, distribution, metabolism, and elimination [42,44,295]. Advancements in nanomaterials-based targeted drug delivery exhibit several benefits, such as examination of the drug sites with the help of imaging agents on the drug carrier, transportation of the macromolecule drugs, therapeutic methods for different therapies, delivery of multiple drugs simultaneously, improved drug delivery of hydrophobic drugs, and targeted drug delivery to a specific cell, tissue, or organ [43,44,60,296]. To serve these benefits, CDs play a substantial role as a nanocarrier in the development of novel drug delivery systems. When a target-sensitive biomolecule is modified at the surface of CDs, CDs-loaded drug delivery systems can specifically target the cancerous or unhealthy cells, by restricting their penetration into normal cells [297,298,299].

Solvothermally synthesized red emissive CDs were used as anti-cancer nanocarriers for the delivery of doxorubicin, an anticancer drug [300]. In this study, doxorubicin-loaded CDs were aimed to penetrate the cancer stem cell nuclei. Upon exposure of HeLa cells with doxorubicin-loaded CDs, the cell viability was found to be reduced to almost 21%, which was quite significant in comparison to the cell viability of HeLa cells (i.e., 50%) when exposed to doxorubicin without CDs. Additionally, it was found that the doxorubicin-loaded CDs have the ability to eradicate the cancer stem cells, hence they could also act as excellent therapeutic agents [300]. In a study by Kong et al., it was reported that the doxorubicin-loaded CDs can also be effectively delivered in MCF-7 breast cancer cells for human breast cancer therapy, owing to their excellent biocompatibility, and high cellular uptake [301].

**Figure 24:**
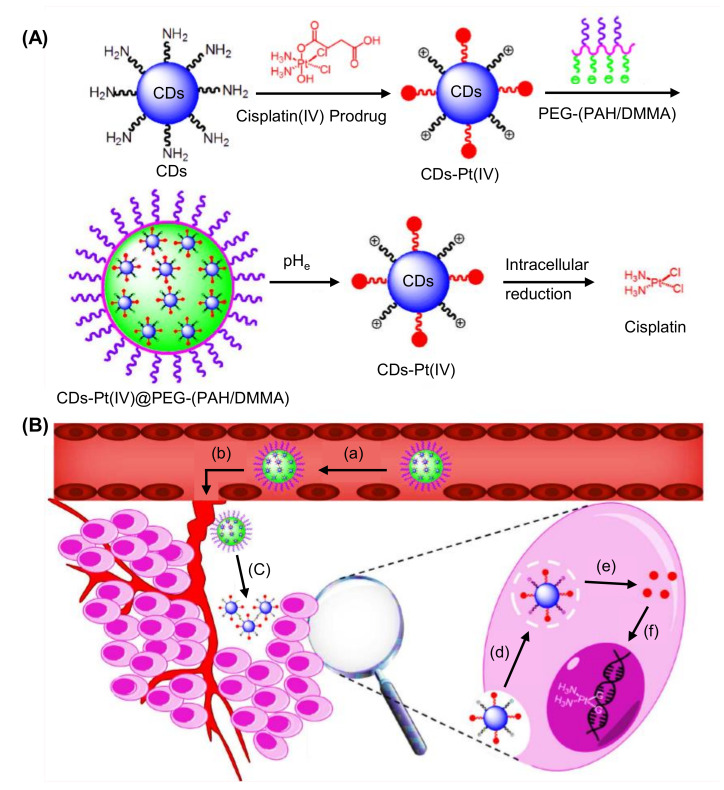
Graphical representation of (**A**) the synthesis and (**B**) the drug delivery system of a charge-convertible CD-based nanocarrier, i.e., CDs–Pt(IV)@PEG-(PAH/DMMA). Reprinted from [302], with permission from American Chemical Society, 2016. DMMA: dimethyl maleic acid; PAH: polyacrylamide hydrochloride; PEG: polyethylene glycol; pH_e_: tumor extracellular pH. (a): Prolonged circulation time; (b) enhanced permeation and retention effect; (c) response to tumor extracellular pH; (d) endocytosis; (e) reduction; (f) DNA binding.

As depicted in Figure 24, using cisplatin(IV) prodrug-incorporated charge-convertible CDs, Feng and co-workers designed a tumor extracellular microenvironment-reactive drug nanocarrier, where an anionic polymer containing polyethylene glycol (PEG) modified polyacrylamide hydrochloride (PAH) and dimethyl maleic acid (DMMA), i.e., PEG-(PAH/DMMA), was coupled with CDs-loaded cisplatin(IV) prodrug (CDs-Pt(IV)) to obtain CDs-Pt(IV)@PEG-(PAH/DMMA), which could result in a captivating charge conversion into a cationic polymer under slightly acidic tumor extracellular microenvironment [302]. This could lead to the liberation of CDs-Pt(IV) in the reductive cytosol via a strong electrostatic repulsion. Under tumor extracellular microenvironment, the in vitro studies revealed the excellent therapeutic effects of CDs-Pt(IV)@PEG-(PAH/DMMA), whereas the in vivo studies confirmed the least adverse effects and the strong tumor-inhibition capacity of CDs-Pt(IV)@PEG-(PAH/DMMA) that suggests its potentiality to be effectively used as a nanocarrier in drug delivery [302].

### 8.2. Role of CDs in Gene Delivery or Gene Therapy

Usually, a tumor-suppressor gene is incorporated into the tumor cells inhibiting growth in gene therapy or gene delivery. Nevertheless, nanomaterials like CDs have proven efficient application as nanocarriers/gene carriers in gene delivery or gene therapy, owing to their excellent biocompatibility and non-toxicity [303,304,305,306]. Nowadays, the highly transfection efficient CDs are being extensively employed for delivering the nucleic acids such as small interfering RNA (siRNA), noncoding RNAs, and DNA, since the CDs are capable enough to deliver genes via endocytosis, macro-pinocytosis, as well as phagocytosis [43,44,307].

**Figure 25:**
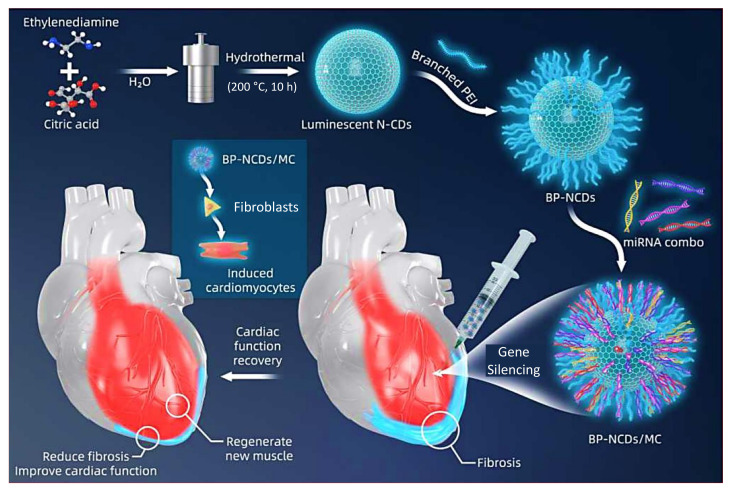
Synthesis of BP-NCDs and their application as a gene carrier for miRNA-combo delivery [308].

Viruses such as lentivirus and retrovirus are broadly exploited as the gene vectors for the direct conversion of fibroblasts into cardiomyocytes. As described in Figure 25, using branched polyethyleneimine-functionalized nitrogen-doped CDs (BP-NCDs) as a non-viral vector, Yang et al. developed a gene delivery strategy for micro-RNAs (miRNAs)-combo (MC) delivery through electrostatic interaction [308]. In this study, it was observed that the fibroblasts, when reprogrammed, were converted into the upregulated cardiomyocyte genes due to the fibroblast gene inhibition, as well as by virtue of the formation of the BP-NCDs/MC nano-conjugates. This suggests the significance of BP-NCDs/MC nano-conjugates as the potent gene carriers in gene delivery for cardiac injury [308].

In a study by He and team, hydrothermally prepared polymeric CDs were employed for enhancing the transfection efficiency in gene delivery [309]. Herein, the as-prepared polymeric CDs could serve as multifunctional gene vectors with excellent biocompatibility, low cytotoxicity, and strong transfection efficiency, by carrying out the luciferase assay in HeLa cells. The as-synthesized polymeric CDs displayed an effective condensation of a positively charged DNA and could prevent the DNA from degrading. It was inferred that the CDs could show higher cell viability, enhanced serum tolerance, and nearly 2000-fold more transfection efficiency, when compared to their polymeric subunits of polyethyleneimine, which implies their prominent role in gene delivery [309].

Wang et al. demonstrated that the delivery of siRNA could be accomplished using polyethyleneimine-adsorbed CDs-based nanocarrier (CDs@PEI) for human gastric cancer therapy [310]. Herein, the direct synthesis of CDs was carried out through the microwave assisted pyrolysis of citric acid, followed by the formation of CDs@PEI conjugates via an electrostatic interaction between the positively charged PEI and the negative charged CDs. Subsequently the siRNAs were added to the CDs@PEI to form siRNA/CDs@PEI [310]. By employing cell cycle analysis, apoptosis assay, Western blotting, and reverse transcription polymerase chain reaction (RT-PCR), the biological influence, cellular uptake, and the gene transfection efficiency of siRNA/CDs@PEI were investigated. From the analysis, it was revealed that the siRNA could readily adhere to the CDs@PEI surface that could lead to the enhancement in the gene delivering capacity. Thus, it can be stated that CDs can act as versatile transmembrane nanocarriers to efficiently deliver either drug, gene, or both, especially in cancer gene therapy [310].

## 9. Applications of CDs in Photodynamic / Photothermal Therapy

Apart from the above-discussed health care applications of CDs, they have also shown significant promise in the area of phototherapies, such as photothermal therapy and photodynamic therapy, owing to their enhanced photostability, high water solubility, and inimitable optical characteristics. Both of these photothermal and photodynamic therapies are non-invasive therapeutic treatments, where the irradiating light is converted into heat by virtue of various photosensitizers, as well as into reactive oxygen species, such as ^1^O_2_, O_2_^•−^, and •OH [44,58,59,84].

### 9.1. Role of CDs in Photodynamic Therapy

Photodynamic therapy has attracted researchers’ interest due to its non-invasive nature and excellent practicability to eliminate tumor cells with less adverse effects, when compared to the conventional cancer treatments. This therapy has very low phototoxic effects on skin, and can cause minimal destruction to the marginal tissues. Moreover, it exhibits very negligible resistance to drugs [311,312,313,314,315]. In this therapy, the accumulation of photosensitizing agents in cancerous cells, and its consecutive irradiation generates reactive oxygen species originating from intracellular oxygen. This results in the necrosis of adjacent malignant cells, and cell death [316,317].

An ideal photosensitizing agent should be photostable, and non-toxic, as well as it should exhibit strong light absorption, high yield for the production of reactive oxygen species. More importantly, it should be effectively taken up by the cancerous tissues or cells [318,319]. Since photosensitizers are highly hydrophobic, nanomaterials like CDs with high hydrophilicity can be combined with them to achieve cellular penetration for the desired bio-application. CDs are employed as potent imaging-oriented therapeutic agents for photodynamic therapy, owing to their large two-photon absorption property, high resistance to photobleaching, chemical inertness, robustness, and high aqueous solubility [319,320,321,322].

Photosensitizers like methylene blue (MB) has been broadly exploited to develop effective photodynamic therapy, although the limitation of using MB individually resides in its hypochromic effect produced due to its binding with DNA in vivo, and self-agglomeration [323]. Therefore, such photosensitizers, when combined with carbon-based nanomaterials like CDs, could display low cytotoxicity, as well as excellent biocompatibility to introduce novel photodynamic therapy. Additionally, it was reported that they exhibit significant resistance to intervention from DNA interaction, agglomeration, and reduction. In a study by Xu et al., the MB-CDs demonstrated convincing ability for the photodynamic therapy, both in vivo as well as in vitro [323].

**Figure 26:**
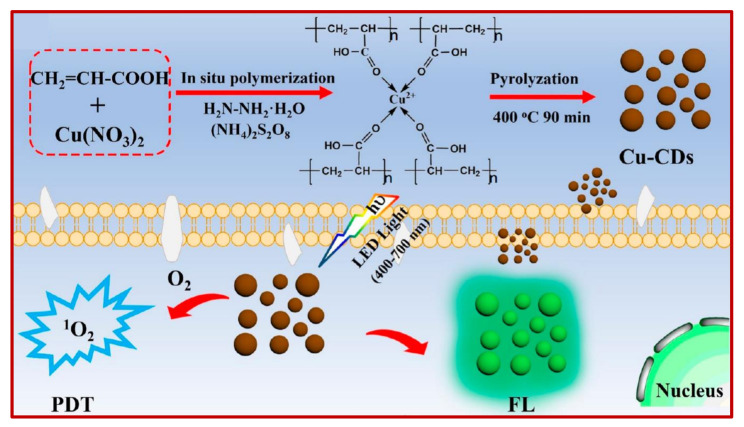
Graphical representation of the preparation of Cu-CDs, and their imaging-regulated application in photodynamic therapy. Reprinted from [324], with permission from American Chemical Society, 2019. FL: fluorescence; PDT: photodynamic therapy.

As shown in Figure 26, the copper-doped CDs (Cu-CDs) synthesized from a copper complex of polyacrylic acid via coordination between the Cu^2+^ ions and the ‒COOH groups, can be employed as an imaging-guided therapeutic agent for photodynamic therapy, as well as for the optical imaging of human neuroblastoma cells and human cervical cancer cells, due to their negligible cytotoxicity, strong fluorescence intensity, and high aqueous solubility [324]. The as-prepared Cu-CDs could exhibit considerable photoinduced cytotoxicity, and a QY of ^1^O_2_ of about 36%. Moreover, the Cu-CDs could efficiently inhibit the growth of human neuroblastoma cells and human cervical cancer cells [324].

Wu and team prepared CDs-decorated platinum porphyrin (CDs@PtPor) nanohybrids for the implementation of a photodynamic therapy for cancer treatment [311]. Herein, the production of CDs@PtPor was achieved via the electrostatic interaction between the negatively charged CDs and the tetraplatinated PtPor conjugates, where the CDs@PtPor could inherit the anti-cancer activity of porphyrin, as well as the fluorescence property of CDs. Due to this synergism, the CDs@PtPor nanohybrids could demonstrate very high photodynamic therapeutic activity, in comparison to the PtPor, which implies that the as-synthesized CDs@PtPor nanohybrids exhibited much more benefits than the traditional formulations [311]. Nevertheless, these CDs@PtPor nanohybrids could render lesser adverse effects in vitro, and an improvised tumor-inhibition capacity. This can be attributed to the increased capacity of CDs@PtPor nanohybrids for the generation of singlet oxygen species [311]. In another work, the synthesis of porphyrin-based CDs (TPP CDs) was carried out using chitosan and mono-hydroxyphenyl triphenylporphyrin (TPP) as precursors by applying the hydrothermal technique [325]. The as-formulated photodynamic TPP CDs exhibited excellent hydrophilicity, strong photostability, and acquired the ability for cellular penetration, as well for the production of cytotoxic singlet oxygen. In vivo experiments revealed that these TPP CDs could inhibit the growth of cancer cells without any adverse effects, demonstrating their potentiality for the enhanced photodynamic therapy in cancer treatment [325].

**Figure 27:**
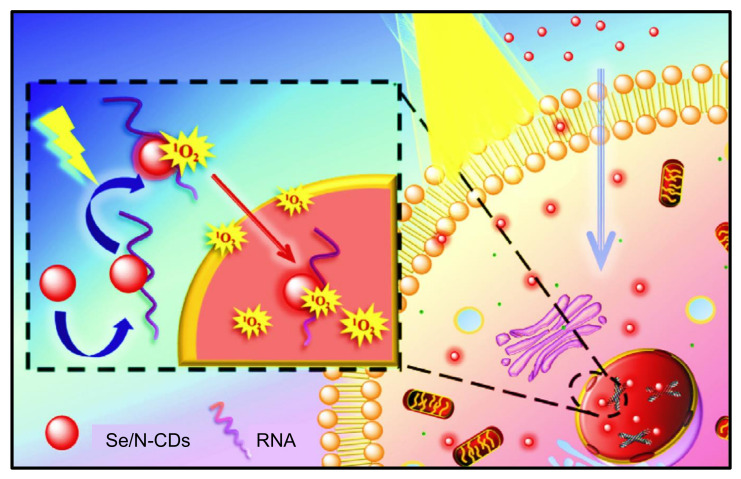
Depiction of an increased photodynamic therapeutic activity by photo-induced Se/N-CDs targeted with RNA. Reproduced from [326], with permission from Elsevier, 2019.

Considering the limitations of photodynamic therapy in vivo due to their restricted diffusion distance of cytotoxic reactive oxygen species (ROS) in the cell and short lifetime, Xu et al. synthesized selenium and nitrogen co-doped CDs (Se/N-CDs) for improvised photodynamic therapy [326]. Applying cell staining with RNA probes, isothermal titration microcalorimetry, and digestion procedures, it was found that the Se/N-CDs could selectively bind to RNA. As depicted in Figure 27, ROS were generated near the nuclear membrane by Se/N-CDs, and upon irradiation of light, this led to the disintegration of the nuclear membrane that could allow the uptake of more Se/N-CDs. As a consequence, the transformation ability of photosensitization was enhanced. Using Se/N-CDs as the photosensitizing agents could offer an effective inhibition of tumor growth in the nucleus itself [326].

Selecting chitosan and diketopyrrolopyrrole (DPP) as the precursors, He et al. prepared diketopyrrolopyrrole-coupled fluorescent CDs (DPP CDs) via hydrothermal technique, and investigated their role in the photodynamic therapy [313]. The DPP CDs could exhibit excellent biocompatibility, as well as aqueous solubility, besides inheriting the property of DPP for ^1^O_2_ generation. Under laser irradiation at 540 nm, the DPP CDs could successfully achieve tumor growth inhibition, which was confirmed by in vitro as well as in vivo experiments [313].

Li and co-workers proposed a CDs-based drug delivery system for photodynamic therapy, as well as chemotherapy [327]. In this work, a nanocarrier system was formulated by conjugating the mono-(5-BOC-protected-glutamine-6-deoxy) b-cyclodextrin (Glu-b-Cyd) with 5-aminolevulinic acid (5-ALA) and CDs to form 5-ALA/CDs/Glu-b-Cyd complex, which was further loaded with doxorubicin. The treatment of MCF-7 breast cancer cells with doxorubicin/5-ALA/CDs/Glu-b-Cyd could prove that the as-developed nanocarrier system was highly cytotoxic for MCF-7 cells, and could lead to the enhancement of cell apoptosis [327].

**Figure 28:**
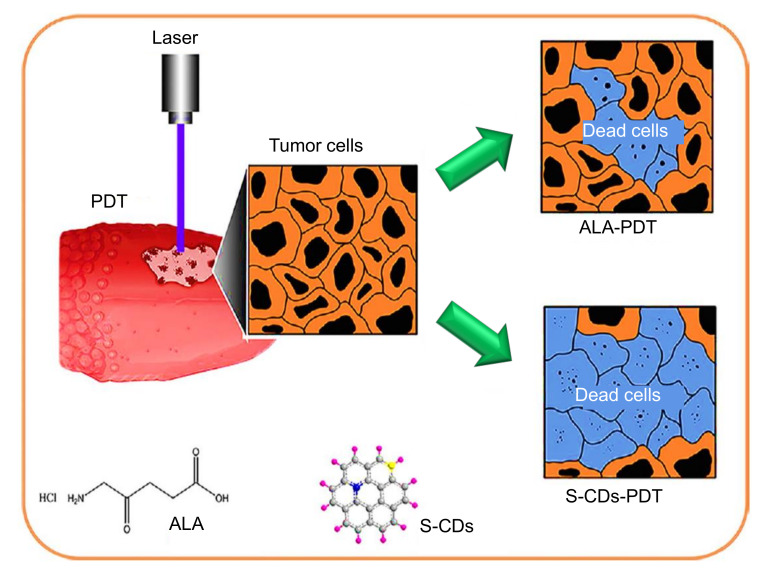
Schematic representation of 5-ALA and S-CDs guided photodynamic therapy. Reproduced from [328], with permission from John Wiley and Sons, 2020. ALA: 5-aminolevulinic acid; PDT: photodynamic therapy.

As portrayed in Figure 28, the sulfur-doped carbon dots (S-CDs) with high yield of ^1^O_2_ were employed as photosensitizing agents for enhancing the photodynamic therapy for oral squamous cancer [328]. The S-CDs could readily penetrate tumor cells and caused cell death upon light irradiation. The treatment of UM1 cancer cells with 5-ALA, as well as with S-CDs showed that those cells treated with 5-ALA could exhibit much lower expression levels of apoptotic proteins than those cells treated with S-CDs at the same concentration. This implies the effectiveness of S-CDs to improvise photodynamic therapy for oral cancer.

### 9.2. Role of CDs in Photothermal Therapy

Over the last few years, just like photodynamic therapy, the photothermal therapy has also aroused widespread interest, and it has been well-established for the treatment of various cancer types [329,330]. This therapy involves the use of photothermal agents for the generation of heat through the absorption of energy in order to achieve cell death. The instrumentation of this therapy includes an NIR absorptive material for the induction of hyperthermia to attain cell death or irreversible cell destruction. The NIR light possesses negligible phototoxicity, and an intensive cell permeation ability. When the photothermal therapeutic agents are combined with the bioimaging, they can monitor the therapeutic response in real-time, by accurately recognizing the location and size of tumor. This is quite significant for developing the improvised, and potent therapeutic strategies [319,331,332,333,334,335,336,337,338]. CDs play a substantial role as photothermal agents, because of the reasons that (i) they are comprised of abundant π electrons, and work equivalently to the free electrons of metallic nanomaterials, (ii) they can result in tremendous temperature changes under irradiation, and (iii) they exhibit photothermal conversion efficiency [43,60,335]

Geng et al. used nitrogen-rich polymer of branched polyethylenimine (BPEI) and 1,3,6-trinitropyrene (TNP) as precursors for synthesizing the nitrogen and oxygen co-doped CDs (N-O-CDs) [339]. Owing to the high photothermal conversion efficiency and low power density, the N-O-CDS were employed as photothermal therapeutic agents in vitro and in vivo, and as fluorescence probes for bioimaging. These N-O-CDs could display outstanding biocompatibility, high photostability, and strong optical absorbance in the near infrared (NIR) region. In vitro and in vivo studies demonstrated that the N-O-CDs could achieve 100% efficacy for tumor cell damage without causing any destruction to the normal healthy cells, which indicates the ability of N-O-CDs for the effective photothermal therapy of cancer [339].

**Figure 29:**
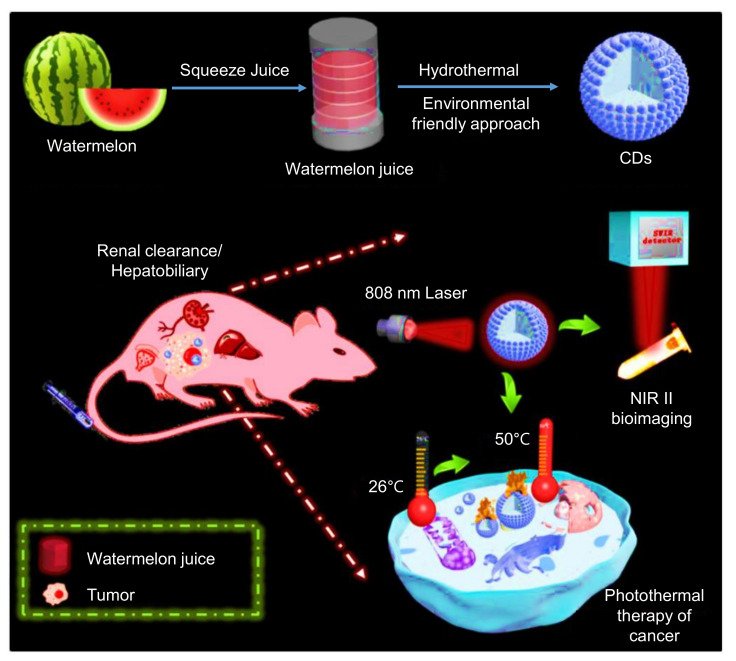
Graphical representation of the preparation of the NIR-II emissive CDs for the photothermal therapy of cancer, and rapid renal clearance NIR-II bioimaging. Reprinted from [340], with permission from American Chemical Society, 2019.

Figure 29 displays the hydrothermal synthesis of NIR-II emissive CDs from watermelon, and their applications in photothermal therapy of cancer, as well as for the rapid renal clearance NIR II bioimaging [340]. The as-synthesized CDs possessed excellent biocompatibility, strong photothermal conversion efficiency upon laser irradiation at 808 nm, high QY, rapid renal clearance, and high photostability. These features of CDs could serve them as the efficient candidates for in vitro and in vivo photothermal therapy and bioimaging applications [340].

Bao et al. investigated the role of S,N-CDs both in bioimaging, as well as in photothermal therapy in vivo [341]. For the solvothermal synthesis of S,N-CDs, dimethylsulfoxide (DMSO), urea, and citric acid were employed as the precursor molecules for providing sulfur, nitrogen, and carbon sources, respectively. Targeting and biodistribution of S,N-CDs could enhance their real therapeutic efficiency. It was found that the S,N-CDs could be easily incorporated into the cancer cells through passive targeting, unlike other nanomaterials that are eliminated via renal excretion [341]. These S,N-CDs could exhibit strong absorbance at NIR region, high photothermal conversion efficiency and could demonstrate their excellence in photothermal therapy, photoluminescence imaging, as well as photoacoustic imaging [341]. Permatasari and coworkers prepared urea-derived pyrrolic-nitrogen containing CDs (N-CDs) via the microwave-assisted hydrothermal technique [342]. These N-CDs possessed strong absorption at NIR region and could be efficiently applied as multifunctional nanocarriers, i.e., for photothermal therapy, drug delivery, and bioimaging [342].

**Figure 30:**
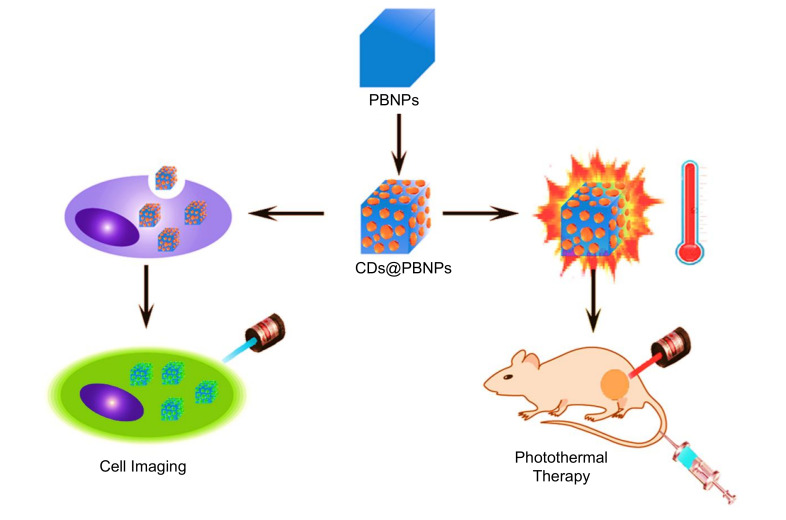
Display of CDs@PBNPs synthesis, and their contribution in photothermal therapy and cell imaging. Reprinted from [334], with permission from American Chemical Society, 2017. PBNPs: Prussian blue nanoparticles.

As displayed in Figure 30, Peng et al. introduced a strategy to develop CDs-coated Prussian blue nanoparticles (CDs@PBNPs) with satellite/core pattern, where the citric acid and urea were selected as the capping agent for the PBNPs stabilization, and for the nitrogen source, respectively [334]. The CDs@PBNPs acquired NIR photo absorption, as well as distinguished green photoluminescence emission with high photothermal stability, and strong photoconversion efficiency. The biocompatibility of CDs@PBNPs was confirmed by in vitro and in vivo toxicity studies. This work suggests that the as-prepared CDs@PBNPs can be applied as an effective tumor ablation therapy, as well as for cell imaging [334].

Lee and the group performed the synthesis of N-CDs by regulating the carbonizing acids and the nitrogen source, and used N-CDs in photothermal therapy as well as in photoacoustic imaging [343]. The as-synthesized N-CDs displayed excellent biodegradability, high photostability, and could show strong absorption peak at the NIR region. Moreover, N-CDs could generate high photoacoustic signals, due to their strong absorbance. This is necessary for the in vivo detection as well as for the non-invasive photothermal therapy [343]. Real-time photoacoustic imaging of sentinel lymph nodes was carried out for the evaluation of the effectiveness, and biodegradability of N-CDs as photoacoustic contrast materials, where the results revealed that these N-CDs can pave the way for photoacoustic imaging, as well for the photothermal therapeutic applications [343].

Zhang et al. proposed a strategy for cancer treatment by combining the photothermal therapy with photodynamic therapy. In their study, they prepared a nanocomposite by coupling the black phosphorus quantum dots (BPQDs) with iron oxide CDs (Fe_3_O_4_-CDs) nanoparticles, and formed genipin [GP]-polyglutamic acid [PGA]-Fe_3_O_4_-CDs@BPQDs [344]. This nanocomposite exhibited high photodegradability, excellent biocompatibility, and showed a strong optical absorbance at NIR region. Moreover, they could effectively inhibit cancer cells, owing to the synergism offered by photodynamic and photothermal therapy through an NIR laser. This was confirmed by the in vitro and in vivo experiments [344].

**Figure 31:**
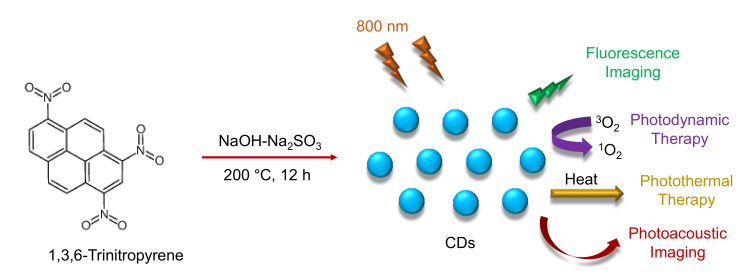
Preparation of TNP-derived CDs, and their versatile role in bioimaging and therapy development. Adapted from [345], with permission from John Wiley and Sons, 2018.

Lan and co-workers carried out the hydrothermal synthesis of CDs using sodium sulfite (Na_2_SO_3_) and 1,3,6-trinitropyrene, and employed them as the single nanocarrier platform for fluorescence imaging, photoacoustic imaging, photodynamic therapy, as well as photothermal therapy in cancer treatment (Figure 31) [345]. The as-prepared CDs could lead to the production of ^1^O_2_ via a two-photon excitation phenomenon, and could emit a strong fluorescence. Upon laser irradiation, they could demonstrate an excellent biocompatibility and high photothermal conversion efficiency [345].

As far as the other health care applications of CDs are concerned, they have also been significantly employed for the scavenging of reactive oxygen species (ROS). Huang et al. hydrothermally prepared sulfur-selenium-doped CDs (S,Se-CDs) with high fluorescence QY [346]. The S,Se-CDs could show the tendency for scavenging ROS, where their antioxidant property could be attributed to the presence of ‒SH and Se‒SH groups on the surface of CDs. In this study, the as-prepared CDs could penetrate into the cells for improving the removal efficiency to ROS [346]. In another study, Luo and co-workers synthesized Se-CDs to scavenge ROS, and applied them for effectively alleviating the secondary injury in traumatic spinal cord injury [347], whereas Wang et al. demonstrated the use of hydrothermally synthesized CDs derived from glutathione and citric acid, for efficient intracellular ROS scavenging to ameliorate the lipopolysaccharide induced inflammation in macrophage [348]. Das and team synthesized CDs from green chili extract by using microwave irradiation technique, where the CDs exhibited the ability for in vitro and in vivo scavenging of ROS, and could deliver a control over ROS scavenging enzyme gene expressions through downregulation [349].

## 10. Summary, Main Challenges, and Future Prospects

In this comprehensive review article, we have described the classification of CDs, their electrical and optical properties, their methods of synthesis, as well as their most commonly employed characterization techniques. From the reviewed studies, the recent trends of CDs, their versality, and substantial roles can be realized for a wide range of health care applications, ranging from electrochemical biosensing, optical biosensing, bioimaging, to gene delivery, drug delivery, photodynamic therapy, and photothermal therapy.

CDs are meticulously developed carbon-based nanomaterials, which have picked up a significant attention and an incredible interest in the field of nanotechnology and biomedical science, which is merely due to their highly considerable and exclusive electrical, optical, as well as biological attributes; including but not limited to their enhanced electron transferability, photobleaching and photo blinking effects, high photoluminescent quantum yield, fluorescence property, resistance to photo-decomposition, increased electrocatalytic activity, good solubility in aqueous media, excellent biocompatibility, long-term chemical stability, cost-effectiveness, negligible toxicity, and large effective surface-area-to-volume ratio. Furthermore, CDs can be readily functionalized owing to the abundant functional groups present on their surface, and they also exhibit remarkable sensing features such as specific, selective, and multiplex detectability. In addition, the physicochemical characteristics of CDs can be easily tunable based on their intended usage or application.

Despite the diverse types of CDs, their fluorescent QY is lower than the semiconductor QDs. This implies that further research should be carried out in a direction to understand the detailed fluorescent mechanism of CDs. Nonetheless, several issues still reside in the practicability of CDs such as synthesizing them with reproducibility and uniformity, in spite of their abundant synthesizing strategies. Since the size distribution of CDs plays a major role in determining their fluorescence properties and toxicity, inadequate size distribution of CDs can be one of the reasons for their limitations in certain bio-applications, especially in vivo. Insufficient reproducibility of CDs in terms of their size, QY, fluorescence intensity, etc., hampers their commercial mass production. Introduction of strategies for their bulk production is necessary, which can be made possible by developing sustainable, economic, efficient, and facile approaches. For the biosensing applications of CDs, the majority of the reported sensors focus on the detection of single analytes. Henceforth, investigations should be made to design CDs-based biosensors for the concurrent detection of multiple target analytes. Although various research works have reported that the CDs are being efficiently employed in various fields, they are still limited to exhibit high quality. An additional surface functionalization is therefore required for serving and expanding their biological applications. However, it is speculated that the new-generation CDs would convincingly address these challenges for suitable healthcare applications in the upcoming future.

## Figures and Tables

**Table 1:** CDs-based electrochemical biosensors reported (2018 onwards) for healthcare applications.

Electrode	Nanomaterials	Receptor Type	Receptor	Target Analyte	Detection Technique(s)	Specimen	Linear Range	Detection Limit	Reference
ITO	CDs-PMMA	Antibody	Anti-TNFα	TNFα	Amperometry	Buffer; human blood	0.05–160 pg mL^−1^	0.05 pg mL^−1^	[238]
SPCE	CDs/ZnO/PANI	Nucleic acid	ss-DNA probe	*E. coli* O157:H7	DPV	Water samples	1.3 × 10^−18^–10 × 10^−12^ M	1.3 × 10^−18^ M	[239]
SPGE	CDs	Nucleic acid	ds-DNA probe	Target DNAs	DPV	DNA isolated from peripheral leucocytes	0.001–20 µM	0.16 nM	[240]
SPCE	CDs	Nucleic acid	Aptamer probe	17 β- estradiol	EIS	Water samples	1 × 10^−7^–1 × 10^−12^ M	0.5 × 10^−12^ M	[241]
Graphite electrode	CDs/PFTBDT	Enzyme	Laccase	Catechol	Amperometry	Water samples	1.25–175 µM	1.23 µM	[242]
-	CoCu-ZIF@CDs	Cells	B16-F10 cell-targeted aptamer strands	B16-F10 cells	EIS	Human B16-F10 living cells	1 × 10^2^–1 × 10^5^ cells mL^−1^	33 cells mL^−1^	[243]
GCE	g-C3N4/N-CDs	MIP	EPI imprinted polymer	EPI	CV; EIS	Human urine samples	1 pM–1 nM	0.3 pM	[244]
PGE	ABSA/CDs	MIP	FA imprinted polymer	FA	CV; DPCSV	Pharmaceuticals; human urine samples	2.2–30.8 ng mL^−1^	2.02 ng mL^−1^	[245]

Abbreviations: ABSA: p-amino benzene sulphonic acid; CDs: carbon dots; Co: cobalt; Cu: copper; CV: cyclic voltammetry; DNA: deoxyribonucleic acid; DPCSV: differential pulse cathodic stripping voltammetry; DPV: differential pulse voltammetry; *E. coli*: *Escherichia coli*; ds: double stranded; EIS: electron impedance spectroscopy; EPI: epinephrine; FA: folic acid; GCE: glassy carbon electrode; g-C_3_N_4_: graphitic carbon nitride; ITO: indium tin oxide; MIP: molecularly imprinted polymer; N-CDs: nitrogen doped CDs; PANI: poly-aniline; PFTBDT: poly[1-(5-(4,8-bis(5-(2-ethylhexyl)thiophen-2-yl)benzo[1,2-b:4,5-b′] dithiophen-2-yl) furan-2-yl) -5-(2-ethylhexyl)-3-(furan-2-yl)-4H thieno[3,4-c]pyrrole-4,6(5H)-dione]; PGE: pencil graphite electrode; PMMA: polymethyl methacrylate; SPCE: screen printed carbon electrode; SPGE: screen printed gold electrode; ss: single stranded; TNFα: tumor necrosis factor α; ZIF: zeolite imidazole framework; ZnO: zinc oxide.

**Table 2:** CDs-based fluorescence biosensors reported (2018 onwards) for healthcare applications.

Nanomaterials	Target Analyte	Specimen	Linear Range	Detection Limit	Reference
CDs	Ferricyanide	Real water samples	5–100 µM	1.7 µM	[269]
N-CDs	Fe^3+^; ATP	Water samples; Human serum	0–350 µM; 0.01–450 µM	0.01 µM; 0.005 µM	[270]
CDs	Diazinon; Amicarbazone; Glyphosphate	Fruit samples	0.25–5000 ng mL^‒1^; 0.5–5000 ng mL^‒1^; 2–5000 ng mL^‒1^	0.25 ng mL^‒1^; 0.5 ng mL^‒1^; 2 ng mL^‒1^	[271]
CDs	Fe^3+^	Real water samples	8–80 µM	3.8 µM	[272]
Cu-CDs	Rutin	Pharmaceutical samples	0.1–15 µg mL^‒1^	0.05 µg mL^‒1^	[273]
CDs	TNP	HeLa cells	5–1000 µM	0.5 µM	[274]
CDs	Pretilachlor	Soil samples	5.7–61.5 µM	2.9 µM	[275]
CDs-AuNCs	Dopamine	Human serum samples	5‒180 nM	2.9 nM	[276]
N,S-CDs	Ascorbic acid	Fruit samples	10–200 µmol L^‒1^	4.69 µmol L^‒1^	[277]
CDs@EDTA	Cr(IV); Ascorbic acid	Real water samples	10 nM–50 µM; 0.1–400 µM	10 nM; 0.1 µM	[278]
CDs	Fe^3+^; Pyrophosphate	Tap water samples; Human urine; Human serum	1–60 µM; 0.1–120 µM	0.28 µM; 0.032 µM	[279]
CDs	Norfloxacin; Ciprofloxacin; Ofloxacin; Histidine	Pharmaceutical tablets; Milk samples	0.05–50 µmol L^‒1^; 0.2–25 µmol L^‒1^; 0.4–10 µmol L^‒1^; 0.05–10 µM	17 nmol L^‒1^; 35 nmol L^‒1^; 65 nmol L^‒1^; 35 nM	[280]
CDs	Hg^2+^	Lake water samples; Human serum	0.50–20 μM	12.4 nM	[281]

Abbreviations: ATP: adenosine triphosphate; AuNCs: gold nanoclusters; CDs: carbon dots; Cr(IV): chromium ions; Cu-CDs: copper-doped CDs; EDTA: ethylene diamine tetra-acetic acid; Fe^3+^: ferric ions; Hg^2+^: mercury ions; N-CDs: nitrogen-doped CDs; N,S-CDs: nitrogen and sulfur co-doped CDs; TNP: 2,4,6- trinitrophenol.
